# Sam68 Is Required for DNA Damage Responses via Regulating Poly(ADP-ribosyl)ation

**DOI:** 10.1371/journal.pbio.1002543

**Published:** 2016-09-16

**Authors:** Xin Sun, Kai Fu, Andrea Hodgson, Eric M. Wier, Matthew G. Wen, Olena Kamenyeva, Xue Xia, Lily Y. Koo, Fengyi Wan

**Affiliations:** 1 Department of Biochemistry and Molecular Biology, Bloomberg School of Public Health, Johns Hopkins University, Baltimore, Maryland, United States of America; 2 Biological Imaging Facility, National Institute of Allergy and Infectious Diseases, National Institutes of Health, Bethesda, Maryland, United States of America; 3 Department of Oncology, School of Medicine, Johns Hopkins University, Baltimore, Maryland, United States of America; 4 Sidney Kimmel Comprehensive Cancer Center, Johns Hopkins University, Baltimore, Maryland, United States of America; The Rockefeller University, UNITED STATES

## Abstract

The rapid and robust synthesis of polymers of adenosine diphosphate (ADP)-ribose (PAR) chains, primarily catalyzed by poly(ADP-ribose) polymerase 1 (PARP1), is crucial for cellular responses to DNA damage. However, the precise mechanisms through which PARP1 is activated and PAR is robustly synthesized are not fully understood. Here, we identified Src-associated substrate during mitosis of 68 kDa (Sam68) as a novel signaling molecule in DNA damage responses (DDRs). In the absence of Sam68, DNA damage-triggered PAR production and PAR-dependent DNA repair signaling were dramatically diminished. With serial cellular and biochemical assays, we demonstrated that Sam68 is recruited to and significantly overlaps with PARP1 at DNA lesions and that the interaction between Sam68 and PARP1 is crucial for DNA damage-initiated and PARP1-conferred PAR production. Utilizing cell lines and knockout mice, we illustrated that Sam68-deleted cells and animals are hypersensitive to genotoxicity caused by DNA-damaging agents. Together, our findings suggest that Sam68 plays a crucial role in DDR via regulating DNA damage-initiated PAR production.

## Abstract 2

The RNA-binding protein Sam68 has unexpected function in the early signaling of DNA damage, and is critical for the activation and regulation of poly(ADP-ribose) polymerase 1 in response to DNA damage.

## Author Summary

Maintaining genome integrity is crucial for all organisms, and failure to do so can lead to fatal diseases such as cancer. Exposure to challenging environments can induce DNA strand breaks or other lesions; thus, rapid and appropriate DNA damage responses (DDRs) need to be in place to detect and repair the damage. Cellular networks use a variety of signaling molecules and post-translational modifications that are crucial for the signaling of DNA breaks to repair machineries. Poly(adenosine diphosphate [ADP]-ribosyl)ation (PARylation) and activation of the enzyme poly(ADP-ribose) polymerase 1 (PARP1) is a post-translational modification that occurs within seconds upon DNA damage detection and triggers downstream DDR signaling; however, it remains obscure whether other molecules, beyond DNA strand breaks, stimulate or control PARP1 activity. We report here that a novel DDR signaling molecule, Src-associated substrate during mitosis of 68 kDa (Sam68), has a crucial function in governing the DNA damage-initiated PARP1 activation and polymers of ADP-ribose (PAR) production. We show that Sam68 is recruited to and significantly overlaps with PARP1 at DNA lesions and that the Sam68-PARP1 interaction is critical for DNA damage-initiated PARP1 activation and PAR production both in vitro and in vivo. Sam68-deleted cells and animals have a diminished PAR-dependent DNA repair signaling and are hypersensitive to genotoxicity caused by DNA-damaging agents. Hence, our data reveal an unexpected function for Sam68 in DNA damage-initiated early signaling and provide a novel mechanism on the activation and regulation of PARP1 in DDR.

## Introduction

DNA damage responses (DDRs) that occur promptly are essential for maintaining genome integrity, which is consistently challenged by internal and external insults [1–6]. Failure to do so can lead to loss of genomic integrity and also cause cancer, immune deficiency, premature aging, and other critical conditions [3,5]. Sophisticated cellular networks, consisting of a variety of molecules and post-translational modifications, are crucial for signaling the presence of DNA strand breaks to repair machineries [3]. In particular, poly(adenosine diphosphate [ADP]-ribosyl)ation (PARylation), catalyzed by the enzymes from the poly(ADP-ribose) polymerase/diphtheria toxin-like ADP-ribosyl transferase (PARP/ARTD) family of proteins [7,8], is one of the earliest events (within seconds) in DDR [9–11]. Previous studies have underscored an indispensable role of PARylation in DNA repair pathways including base excision repair (BER), single-strand break repair (SSBR), homologous recombination (HR), and nonhomologous end joining (NHEJ) [12–17]. Importantly, the elongated and branched structure enables polymers of ADP-ribose (PAR) to serve as a docking platform for the focal assembly of DNA repair complexes, thus orchestrating appropriate DDR signaling cascades [18–26]. For instance, following γ-irradiation, phosphorylation/activation of the proximal checkpoint kinase ataxia telangiectasia mutated (ATM) as well as ATM substrates checkpoint kinase 1 (Chk1) and Chk2 occurs in a PAR-dependent manner [14,27]. As the founding member of PARP/ARTD superfamily, PARP1 (also named ARTD1) is the major enzyme responsible for the rapid and vigorous PAR synthesis triggered by damaged DNA [1,10]. Binding of PARP1 to DNA strand breaks results in conformational changes in PARP1 and elevates its activity [4]. Upon activation, PARP1 vigorously synthesizes and adds ADP-ribosyl polymers to a variety of target proteins, including PARP1 itself [9]. Albeit these important advances in understanding of the critical function of PARylation in DDR, the precise mechanisms of stimulation and regulation of PARP1 catalytic activity during DDR are still obscure. In particular, recent studies showed that DNA strand breaks appear not to be the sole stimulatory factor for PARP1 activation [9,10,28–33], which suggests that a more complicated mechanism or mechanisms could be required to robustly activate and elegantly fine-tune PARP1 activity. Moreover, the inhibition of PARP1/PARylation has emerged as a promising therapeutic approach for treating human cancers and inflammatory diseases associated with impaired DNA repair activities [34,35]. Of note, the current classes of PARP1 inhibitors, either approved by FDA or undergoing clinical trials, are all based on a competitive binding strategy first observed with nicotinamide [36]. Therefore, elucidating the molecular mechanisms of activation and regulation of PARP1 activity in DDR may provide clinical relevance to aid the rational development of new PARP1 inhibitors.

Src-associated substrate during mitosis of 68 kDa (Sam68) is a versatile RNA-binding protein and plays a role in a wide range of cellular processes, including RNA stability, RNA splicing, RNA nuclear export, HIV-1 replication, adipogenesis, neuronal activity, and others [37–47]. We and others recently showed that Sam68 participates in the transcription of certain genes via its interactions with transcription factors [43,48–51], which is in line with the fact that Sam68 binds single- and double-stranded DNA, besides RNA. Moreover, emerging evidence suggests that RNA-binding proteins play critical functions in DNA damage signaling [52–54]. In particular, Sam68 was identified as a PAR-binding partner in cells with DNA damage and proposed as a putative substrate of ATM, ataxia telangiectasia and Rad3-related (ATR), and DNA-dependent protein kinase (DNA-PK) [55,56], which strongly indicates that Sam68 could execute an important function in DDR. Although Sam68, a ubiquitously expressed RNA-binding protein, has been long acknowledged as an almost strictly nuclear protein [38,41], its potential function in the nuclear-initiated signaling pathways, especially DDR, has not been the subject of intense investigation. In this manuscript, we report that Sam68, as a novel signaling molecule in DDR, plays a crucial function in governing the DNA strand break-triggered PARP1 activation and PAR production. Upon DNA damage, Sam68 is recruited to and significantly overlaps with PARP1 at DNA damage sites. Interaction between Sam68 and PARP1 via their N-termini is critical for DNA dependent-PARP1 activation and PAR production in vivo and in vitro. In line with the attenuated PAR-dependent repair signaling, DNA damage is poorly repaired in Sam68-deficient cells and animals in comparison to the Sam68-sufficient controls. As a consequence, Sam68 knockout mice are hypersensitive to genotoxicity caused by γ-irradiation and DNA alkylating agents. Hence, our data reveal an unexpected function for Sam68 in DNA damage-initiated early signaling and provide a novel mechanism on the activation and regulation of PARP1 in DDR.

## Results

### Sam68 Plays a Critical Role in Repairing DNA Strand Breaks

**Fig 1:**
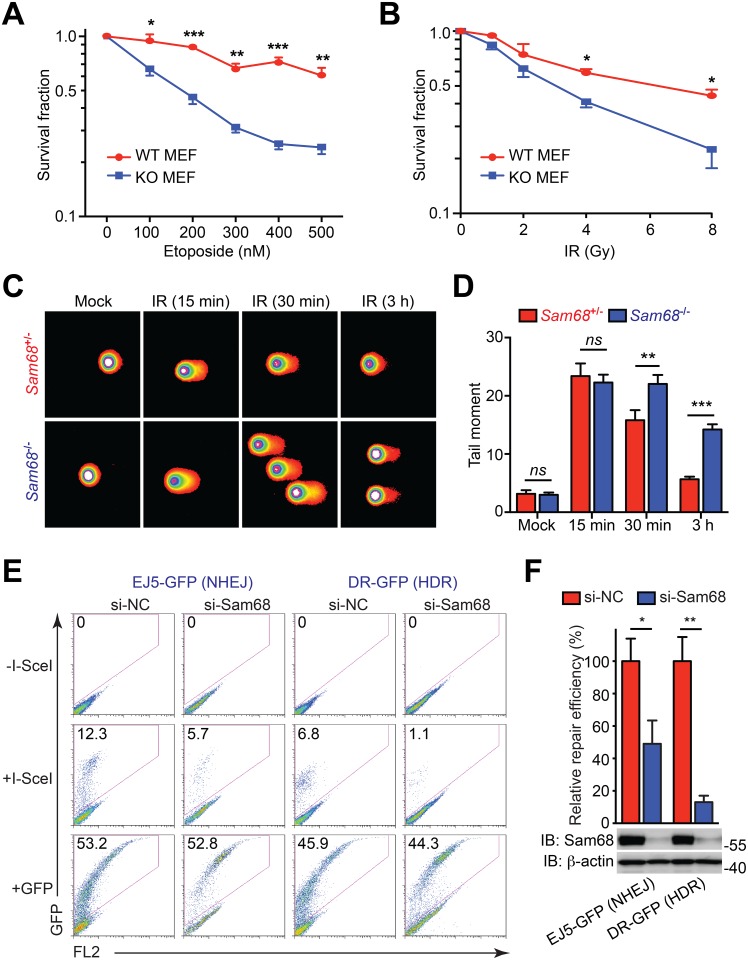
Sam68 is required for repairing DNA strand breaks. (A, B) Survival fraction of wild-type (WT) and Sam68 knockout (KO) mouse embryonic fibroblasts (MEFs) 96 h post treatment with indicated concentrations of etoposide for 20 h (A) or indicated doses of γ-irradiation (IR) (B). (C) Representative microphotographs of alkali comet assay of *Sam68*^+/-^ and *Sam68*^-/-^ thymocytes at indicated time points following 4 Gy of IR or mock-treated. (D) Quantification of tail moments in C, with summarized data from 40–60 cells within 15 random fields for each time point. (E) Flow cytometric detection of effect of Sam68 knockdown on DNA damage repair efficiency. U2OS reporter cell lines specifically designed to repair DNA damage through nonhomologous end joining (NHEJ) and homology-directed repair (HDR), were transfected with nonspecific control (si-NC) or Sam68-specific (si-Sam68) small interference RNA, together with (+) or without (−) I-SceI plasmid, or green fluorescent protein (GFP) control. Shown are representative flow cytometry analyses of the frequency of GFP^+^ cells in indicated reporter cell lines 72 h following transfection. (F) Quantification of relative repair efficiency (normalized to si-NC and I-SceI cotransfected cells) in indicated reporter cell lines, summarized from three independent experiments. The Sam68 knockdown efficiency was examined by immunoblot (IB), with β-actin as a loading control, in indicated reporter cell lines (bottom). Results in (A), (B), (D), and (F) are expressed as mean and standard error of the mean (SEM). ns, nonsignificant difference; *, *p* < 0.05; **, *p* < 0.01; ***, *p* < 0.001 by Student’s *t* tests. Data are representative of at least three independent experiments. Underlying data are shown in S1 Data.

Hypersensitivity to DNA-damaging agents is one of the hallmarks of defective DDR. To address the role of Sam68 in repair of DNA strand breaks, we first examined the effect of Sam68 deletion in clonogenic survival of mouse embryonic fibroblasts (MEFs) following exposure to genotoxic stresses. Sam68 deletion in MEFs led to an increased sensitivity to etoposide (a DNA-damaging agent that inhibits DNA topoisomerase II), γ-irradiation, and H_2_O_2_ compared with wild-type cells (Fig 1A and 1B and S1 Fig). To ascertain whether Sam68 is essential for the completion of DNA repair, we performed single-cell gel electrophoresis-based alkaline comet assays, a sensitive method for detecting DNA strand breaks [57]. No comet tails were observed in mock-irradiated Sam68^-/-^ and Sam68^+/-^ primary thymocytes, suggesting Sam68 deletion does not spontaneously cause DNA damage (Fig 1C and 1D). The vast majority of γ-irradiated thymocytes, in the presence or absence of Sam68, showed prominent comet tail moments, an indicator of DNA damage severity, at 15 min post γ-irradiation (Fig 1C and 1D). The comet tails lessened in a time-dependent manner in Sam68^+/-^ thymocytes, and almost no comet tails were detected at 3 h post γ-irradiation. Strikingly, the comet tails remained prominent in Sam68^-/-^ thymocytes during the same time period (Fig 1C and 1D), which strongly supports an indispensable role of Sam68 in repairing DNA breaks.

DNA double-strand breaks (DSBs) are the most severe form of damage to DNA, and homology-directed repair (HDR) and NHEJ have been proposed as the major mechanisms used to repair DSBs [58]. We sought to directly test whether Sam68 facilitates DNA repair through one or more such specific signaling pathways. To this end, we utilized U2OS cell lines that contain chromosomally integrated green fluorescent protein (GFP) reporters with recognition sites for the rare-cutting endonuclease I-SceI to assess the rates of HDR and NHEJ, as GFP positivity by flow cytometry analysis suggests that repair has occurred in these cells [58]. Upon down-regulation of Sam68 by small interference RNA (siRNA), we observed the repair efficiency of the NHEJ and HDR pathways was reduced to 42.3% and 12.2%, respectively, in comparison to the nonspecific control siRNA (Fig 1E and 1F). The efficiency of Sam68 silencing in the U2OS reporter cell lines was verified by IB (Fig 1F), and Sam68 down-regulation exhibited no substantial effect on transfection efficiency of these cells (Fig 1E). Moreover, ectopic expression of a Sam68 construct that is resistant to siRNA markedly rescued the repair efficiency of the NHEJ and HDR pathways in the Sam68 knockdown cells (S2 Fig). Together, these results suggest that Sam68 deletion causes defective DNA repair, thus resulting in persistent DSBs and hypersensitivity to DNA-damaging agents.

### Sam68 Deficiency Impairs DNA Damage-Initiated PARylation and Signaling Cascade

**Fig 2:**
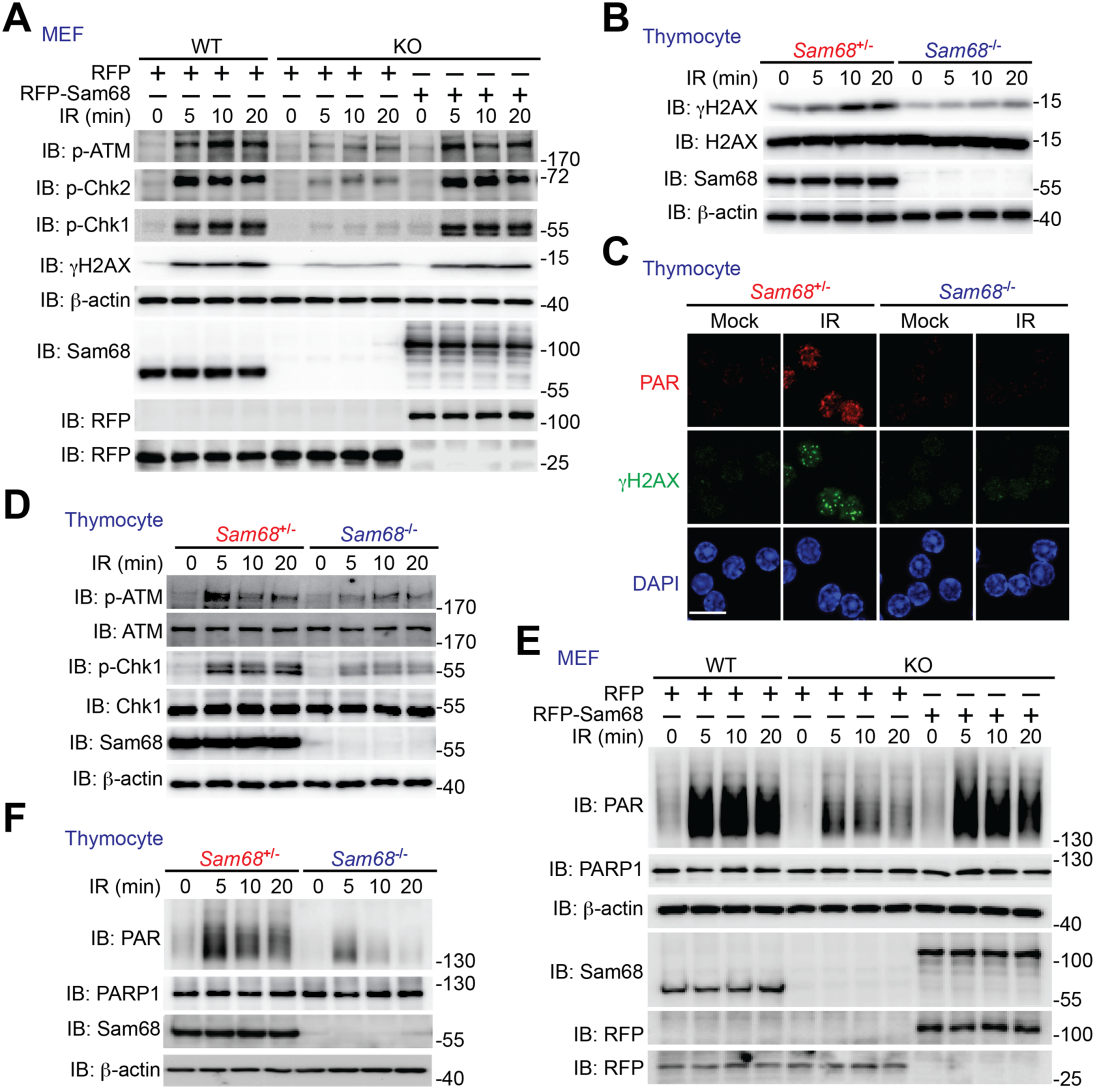
Sam68 deficiency attenuates DNA damage-initiated repair signaling in cell culture. (A, E) WT and Sam68 KO MEFs expressing red fluorescent protein (RFP) or RFP-Sam68 were γ-irradiated (γ-irradiation, IR) at 10 Gy, and whole cell lysates were derived at indicated time points and immunoblotted (immunoblot, IB) for indicated proteins, with β-actin as a loading control. (B, D, F) Primary thymocytes isolated from *Sam68*^+/-^ and *Sam68*^-/-^ mice were γ-irradiated at 4 Gy, and whole cell lysates were derived at indicated time points and immunoblotted for indicated proteins, with β-actin as a loading control. (C) Immunofluorescence micrographs of PAR and γH2AX in thymocytes collected at 15 min post mock- or γ-irradiation (IR, 4 Gy), with nuclei counterstained by 4′, 6-diamidino-2-phenylindole (DAPI). Scale bar, 10 μm.

As Sam68 knockdown has a more profound impact on the efficiency of HDR (Fig 1E and 1F), which is the most important error-free pathway for repairing DSBs, we sought to examine how Sam68 deficiency affects the signaling cascade in response to DSBs caused by γ-irradiation. Phosphorylation of the histone variant H2AX (γH2AX), a DNA damage marker that promotes the recruitment of chromatin-modifying complexes and downstream repair factors [59–61], was robustly increased in Sam68-sufficient MEFs, primary thymocytes, and U2OS cells treated with γ-irradiation (Fig 2A–2C and S3 Fig). In contrast, such DNA damage-triggered γH2AX accumulation was severely attenuated in Sam68-deficient cells (Fig 2A–2C and S3 Fig). Moreover, the γ-irradiation-induced phosphorylation of ATM (which is the kinase of H2AX) and its substrates Chk1 and Chk2, all of which are essential DNA damage signaling transducers [1], was consistently more robust in Sam68-sufficient MEFs, thymocytes, and U2OS cells in comparison to Sam68-deficient cells (Fig 2A and 2D and S3 Fig). These observations further suggest that Sam68 deficiency leads to decreased DNA repair signaling. As DSB-induced ATM phosphorylation is known to rely on the activation of the DNA damage sensor PARP1 and subsequent PAR production [14,27], we examined PAR chain formation in γ-irradiated cells in the presence and absence of Sam68. As expected, PAR chains were rapidly and vigorously built up in Sam68-sufficient thymocytes and MEFs following γ-irradiation (Fig 2C, 2E and 2F). However, while Sam68 deficiency did not affect PARP1 levels (Fig 2E and 2F), γ-irradiation triggered PAR production was diminished in Sam68-deficient cells (Fig 2C, 2E and 2F), which demonstrates that Sam68 is crucial for DNA damage-initiated PAR chain formation. Moreover, supplementing with exogenous Sam68 markedly restored the DNA damage-initiated PAR synthesis and the PAR-dependent phosphorylation of ATM, Chk1, Chk2, and H2AX in Sam68 knockout (KO) MEFs following γ-irradiation (Fig 2A and 2E), highlighting the crucial role of Sam68 in the DSB-triggered PAR production and PAR-dependent signaling to DNA repair.

### DNA Damage Enhances Interaction between Sam68 and PARP1

**Fig 3:**
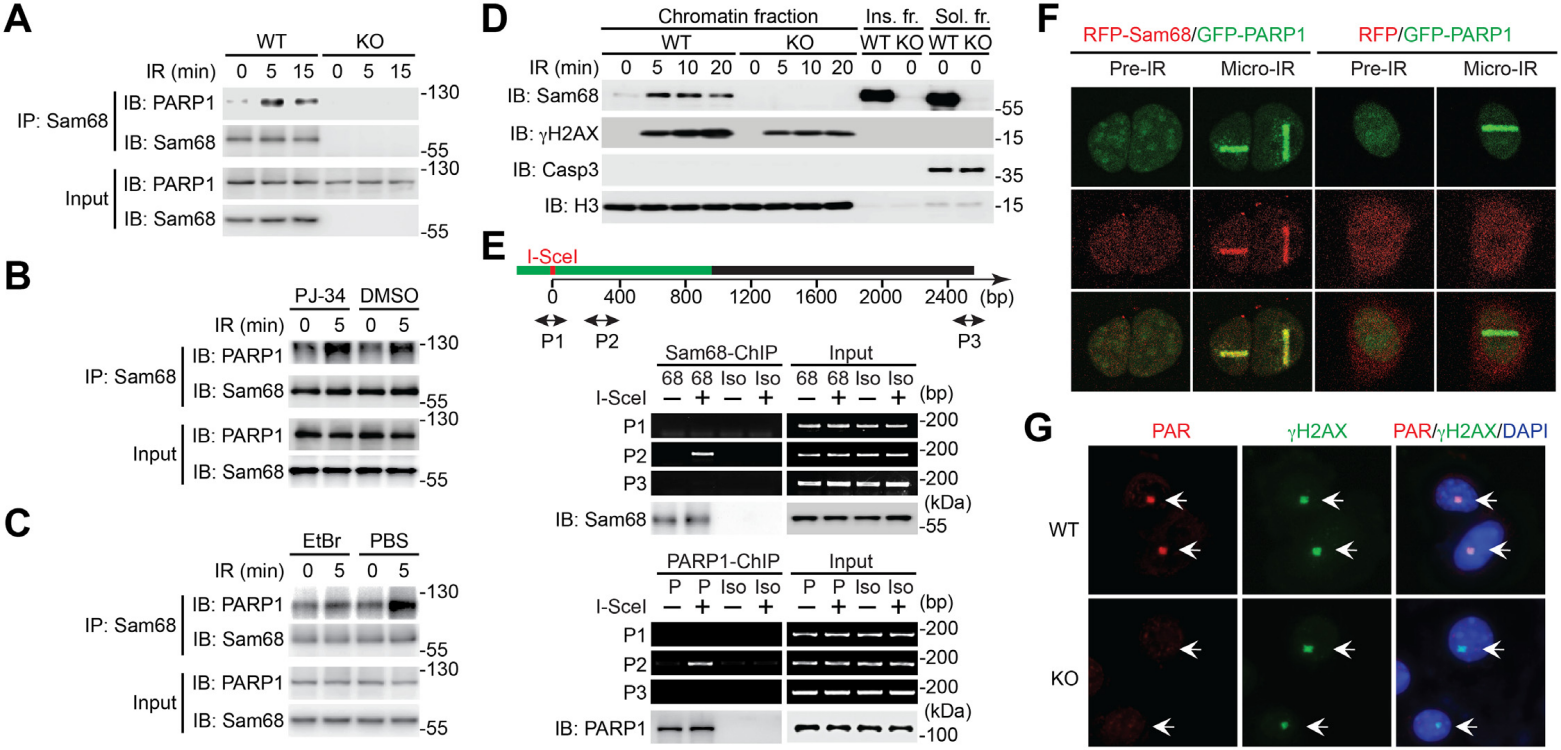
Sam68 is recruited to and regulates PAR production at DNA damage sites. (A) Coimmunoprecipitation showing the inducible Sam68-PARP1 interaction. WT and Sam68 KO MEFs were γ-irradiated at 10 Gy, and whole cell lysates (Input) derived at indicated time points post irradiation were immunoblotted directly or after immunoprecipitated (immunoprecipitation, IP) with Sam68 antibody for indicated proteins. (B) WT MEFs were pretreated with DMSO or PJ-34 (20 μM) for 1h, followed by IR at 10 Gy. Whole cell lysates (Input) derived at the indicated periods post irradiation were immunoblotted directly or after immunoprecipitated with Sam68 antibody for the indicated proteins. (C) WT MEFs were γ-irradiated at 10 Gy. Cells were harvested at the indicated periods post irradiation and lysed in the lysis buffer supplemented with phosphate-buffered saline (PBS) or ethidium bromide (EtBr, 50 μg/ml). The derived whole cell lysates (Input) were immunoblotted directly or after immunoprecipitated with Sam68 antibody for the indicated proteins. (D) Chromatin fractionation assays showing dynamics of Sam68 on damaged chromatin. WT and Sam68 KO MEFs were γ-irradiated at 10 Gy, and the chromatin, soluble (Sol. fr.), and insoluble (Ins. fr.) subcellular fractions were derived at indicated time points post γ-irradiation and immunoblotted for indicated proteins. Casp-3, Caspase-3; H3, Histone H3. (E) Chromatin immunoprecipitation (ChIP) analyses showing Sam68 recruitment to the DNA double-strand break (DSB) at I-SceI site in HDR-GFP U2OS reporter cells. Upper, diagram shows the locations of I-SceI site and PCR products amplified by primer set 1 (P1), P2, and P3 on HDR-GFP chromosome. HDR-GFP U2OS reporter cells were transfected with (+) or without (−) I-SceI plasmid for 20 h, and the nuclear extracts (Input) were derived and subjected to ChIP assays with isotype (Iso), Sam68 (68), or PARP1 (P) antibody. PCR evaluation of the enrichment of Sam68 or PARP1 (as a positive control) adjacent to the DSB using indicated primer sets. Bottom, inputs, and chromatin immunoprecipitants were immunoblotted for Sam68 and PARP1, respectively. (F) Fluorescence micrographs of Sam68 KO MEFs transiently expressing RFP-Sam68 or RFP together with GFP-PARP1, before (Pre-IR) and after laser microirradiation (Micro-IR). (G) Immunofluorescence micrographs of endogenous PAR in WT and Sam68 KO MEFs at 1 min post laser microirradiation, with nuclei counterstained by DAPI. As positive controls, γH2AX form damage foci (indicated by arrows).

Given that Sam68 deletion almost abolished PAR production following γ-irradiation (Fig 2E and 2F), we first performed immunoprecipitation assays to examine whether Sam68 interacts with PARP family proteins in DDR, thus affecting the DNA damage-initiated PAR formation. Indeed, at 5 min post γ-irradiation, Sam68 substantially associated with PARP1 and ATM (Fig 3A and S4 Fig); in contrast, no detectable interaction between Sam68 and PARP2, PARP3, and PARP5a/b was observed under the same conditions (S4B Fig). Given the observed interaction and that PARP1 is the primary nuclear enzyme that transfers PAR chains to various target proteins in response to DNA damage [2], we further characterized the Sam68-PARP1 interaction following DNA damage. The γ-irradiation-triggered Sam68-PARP1 association was almost identical in the cells pretreated with the PARP inhibitor PJ-34 compared to control (Fig 3B), which suggests that PAR chain formation is dispensable for DNA damage-induced Sam68-PARP1 interaction. In contrast, the Sam68-PARP1 interaction in response to γ-irradiation was greatly reduced in the presence of the DNA-binding agent, ethidium bromide (Fig 3C), demonstrating that damaged DNA is critical for the DNA damage-induced Sam68-PARP1 interaction.

### Sam68 Is Recruited to and Regulates PAR Production at DNA Damage Sites

The fact that PARP1 is rapidly recruited to DNA damage sites [62] and damaged DNA enhances the Sam68-PARP1 interaction in DDR (Fig 3A and 3C) led us to assess whether Sam68 can be recruited to DNA lesions where it interacts with PARP1 and regulates PAR formation. As illustrated by chromatin fractionation assays, Sam68 was remarkably enriched in chromatin fractions in MEFs and U2OS cells following γ-irradiation (Fig 3D and S5 Fig). To ascertain whether the damaged chromatin-enriched Sam68 is recruited to DNA lesions rather than generally bound to chromatin, we performed chromatin immunoprecipitation (ChIP) assays to monitor the DNA damage-triggered Sam68 recruitment to the proximity of a DSB that is actively generated at a unique chromosomally integrated I-SceI site in HDR-GFP reporter U2OS cells (Fig 3E). While no association of Sam68 or of PARP1 was detected with chromatin near the I-SceI site in U2OS cells without I-SceI transfection, Sam68 and PARP1 were indeed enriched at the site near the generated DSB in the I-SceI-expressing U2OS reporter cells (Fig 3E). In contrast, we did not detect an enrichment of Sam68 or PARP1 at the site far away from the cut I-SceI site (Fig 3E), which suggests that Sam68 and PARP1 are specifically recruited to the DNA damage site. Moreover, we carried out laser microirradiation microscopy assays to visualize the recruitment of Sam68 to local DNA strand breaks, using MEFs expressing red fluorescent protein (RFP)-tagged Sam68 as well as GFP-tagged PARP1. Consistent with the previous studies [2], accumulation of ectopically expressed GFP-PARP1 at DNA lesions was manifested as cytological discernable foci in laser-irradiated Sam68 KO MEFs (Fig 3F and S6A Fig). Interestingly, RFP-Sam68 formed discrete, cytologically detectable foci, which significantly overlap with the damage foci formed by GFP-PARP1 and endogenous γH2AX, after laser microirradiation in parallel samples all collected at the same time post micro-irradiation (Fig 3F and S6A Fig). In contrast, the RFP control failed to form discrete foci, although GFP-PARP1 formed damage foci as expected, under the same condition, in the cells expressing RFP and GFP-PARP1 (Fig 3F). Of note, endogenous Sam68 and PARP1 also formed discrete DNA damage foci that significantly overlapped with those manifested by γH2AX, after laser microirradiation (S6B Fig), further supporting our assertion that Sam68 localizes at sites of damaged DNA significantly overlapping with PARP1 as well as γH2AX during the cellular responses to DNA damage. Moreover, the GFP-PARP1 fluorescence intensities after laser microirradiation were comparable in wild-type and Sam68-deleted cells (S6C Fig), indicating that Sam68 is not required for PARP1 localization to sites of damaged DNA. Similarly, PARP1 inhibition with Olaparib, compared to the vehicle control, did not affect the recruitment of GFP-PARP1 at sites of damaged DNA (S6D Fig). In contrast, Olaparib treatment substantially augmented, rather than attenuated, the localization of RFP-Sam68 at DNA damage foci in the MEFs expressing RFP-Sam68 and GFP-PARP1 (S6E Fig). These results suggest that Sam68 and PARP1 both localize to sites of damaged DNA independently. To assess the impact of Sam68 on the PAR formation at local DNA damage sites, we carried out immunofluorescence staining assays for PAR production at local DNA strand breaks generated by laser microirradiation in the nucleus in the presence and absence of Sam68. As expected, shortly (1 min) after laser microirradiation, vigorous PAR production was detected locally in the nuclear regions where laser beams were introduced in WT MEFs (Fig 3G). In striking contrast, PAR formation was barely observed in the laser microirradiated regions in Sam68 KO MEFs (Fig 3G), which suggests that Sam68 is crucial for local PAR synthesis at DNA lesions. Collectively, these results demonstrate that Sam68 is recruited to and significantly overlaps with PARP1 at DNA damage sites, where it regulates local PAR production in the cellular responses to DNA damage.

### Sam68 Stimulates PARP1 Catalytic Activity via the Interaction between Their N-termini

**Fig 4:**
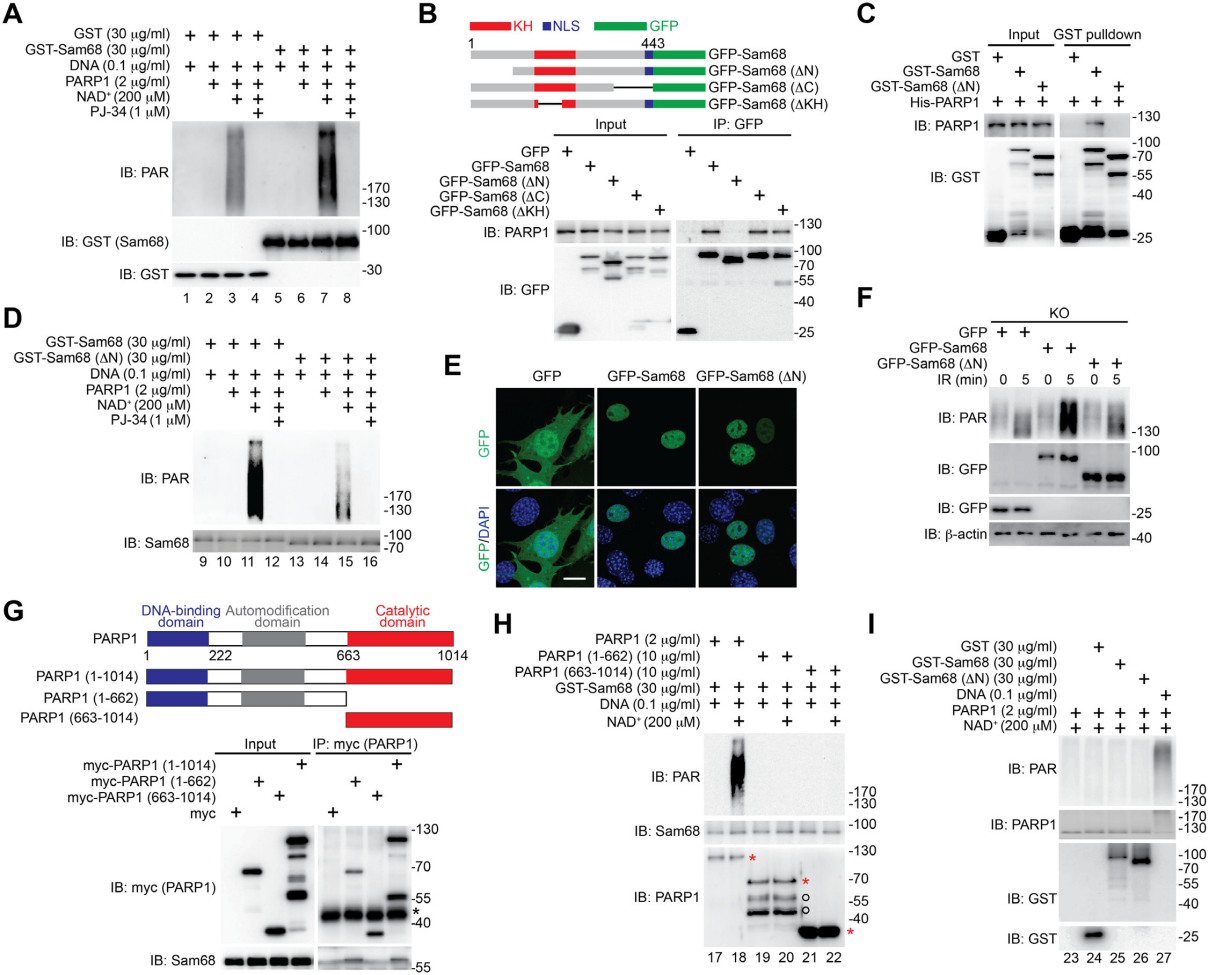
Sam68 enhances the damaged DNA-dependent PARP1 activation and PAR production in vitro. (A, D) Recombinant Sam68, Sam68 (ΔN), or GST control protein was incubated in reaction buffer containing damaged DNA alone or with recombinant PARP1 protein in the presence and absence of NAD^+^, with indicated final concentrations. Where indicated, PARP inhibitor PJ-34 was added to the reaction mixture. The reaction mixture was separated by SDS-PAGE and subjected to immunoblotting with the indicated antibodies. (B) Schematic diagram of the GFP-tagged full-length (residues 1–443) and truncated mutants (ΔN lacks residues 1–102, ΔC lacks 347–443, and ΔKH lacks 165–224) of Sam68. KH, hnRNP K homology; NLS, nuclear localization signal. Whole cell lysates (Input) from HEK293T cells expressing GFP or indicated Sam68 fusion proteins were immunoblotted directly or after immunoprecipitated with GFP antibody for the indicated proteins. (C) Whole cell lysates (Input) containing His-PARP1, GST, GST-Sam68, or GST-Sam68 (ΔN) recombinant proteins were immunoblotted directly, or following GST pulldown, for indicated proteins. (E) Immunofluorescence micrographs of Sam68 KO MEFs expressing GFP, GFP-Sam68, or GFP-Sam68 (ΔN) proteins, with nuclei counterstained by DAPI. Scale bar, 10 μm. (F) Sam68 KO MEFs expressing GFP, GFP-Sam68, or GFP-Sam68 (ΔN) proteins were mock- or γ-irradiated at 10 Gy, and whole cell lysates were derived at 5 min post γ-irradiation and immunoblotted for indicated proteins, with β-actin as a loading control. (G) Schematic diagram of the PARP1 full-length (residues 1–1,014), with indicated DNA-binding, automodification, and catalytic domains, and truncated mutants (residues 1–662 and 663–1,014) of PARP1. Whole cell lysates (Input) from HEK293T cells expressing myc-tagged PARP1, PARP1 (1–662), or PARP1 (663–1,014) protein were immunoblotted directly, or after immunoprecipitation with myc antibody, for indicated proteins. The nonspecific antibody heavy chains are labeled with an asterisk. (H, I) Recombinant proteins were incubated in reaction buffer in the presence and absence of damaged DNA and NAD^+^, with indicated final concentrations. The PARP1, PARP1 (1–662), PARP1 (663–1014) proteins and spontaneously degraded PARP1 (1–662) species are labeled with asterisks and cycles, respectively (H). The reaction mixture was separated by SDS-PAGE and subjected to IB for the indicated proteins.

The findings that Sam68 deficiency attenuates DNA damage-induced global PAR production (Fig 2E and 2F) and focal PAR formation (Fig 3G) and that Sam68 and PARP1 interact and overlap at DNA strand breaks after DNA damage (Fig 3A and 3F) led us to hypothesize that Sam68 governs DNA damage-initiated PARylation via directly stimulating the catalytic activity of PARP1. To test this hypothesis, we performed in vitro PARylation assays using recombinant PARP1 and Sam68 proteins. As expected, damaged DNA-activated PARP1 automodified itself with the addition of PAR moieties from the supplemented nicotinamide adenine dinucleotide (NAD^+^), as indicated by the robust PAR chain formation (Fig 4A and S7A Fig). Strikingly, incubation of recombinant Sam68 protein, compared to the GST control, with PARP1 dramatically boosted PARP1 activation and PAR production in a dose-dependent manner (Fig 4A, compare lane 3 with lane 7, and S7B Fig) in the presence of damaged DNA and NAD^+^. Moreover, in the absence of PARP1, Sam68 recombinant protein was not able to produce a PAR chain with supplemented damaged DNA and NAD^+^ (S7C Fig), indicating that Sam68 per se does not possess the enzymatic activity to transfer ADP-ribosyl polymers. Hence, our results demonstrate that Sam68 simulates PARP1 activation and subsequent PAR production in the presence of damaged DNA and NAD^+^.

We sought to understand the key domain(s) in Sam68 critical for the interaction with PARP1 and the PARP1-stimulatory function using various Sam68 truncates. We detected the association of the full-length, ΔC, and ΔKH truncated Sam68 to endogenous PARP1, but not GFP vehicle (Fig 4B). In contrast, deletion of the N-terminal amino acids 1–102 (ΔN) of Sam68 almost abolished the association of Sam68 to PARP1 (Fig 4B), suggesting a key role of the N-terminus for the interaction between Sam68 and PARP1. Moreover, we carried out pulldown assays using GST-fused full-length and ΔN truncated Sam68 together with PARP1 recombinant proteins. While full-length Sam68 pulled down PARP1, the ΔN truncated Sam68 failed to do so (Fig 4C), which further confirms the critical role of the N-terminus of Sam68 for the Sam68-PARP1 interaction. To examine whether the Sam68 N-terminus-mediated Sam68-PARP1 interaction is important for the PARP1-stimulatory function, we conducted the in vitro PARylation assays, supplementing PARP1 with full-length or ΔN mutant Sam68 recombinant protein. Indeed, the stimulatory effect of Sam68 for PARP1 activation and PAR production was substantially reduced when Sam68 (ΔN) recombinant protein was utilized, in comparison to full-length Sam68 (Fig 4D, compare lane 11 with lane 15). This result underscores an important role of the Sam68 N-terminus in controlling PARP1 activity in vitro. Moreover, to further examine whether the Sam68-PARP1 interaction is functionally important for DNA damage-initiated PAR production in cells, we compared the γ-irradiation triggered PARylation in Sam68 KO MEFs transiently expressing full-length or ΔN truncated Sam68, both of which share strict nuclear localization (Fig 4E). The expression of full-length Sam68, but not GFP control, significantly restored the DNA damage-initiated PAR synthesis in Sam68 KO MEFs (Fig 4F), consistent with our previous observation (Fig 2E). However, ectopic expression of Sam68 (ΔN) mutant failed to restore the γ-irradiation triggered PAR production in Sam68 KO MEFs (Fig 4F). Thus, our results demonstrate that the N-terminus of Sam68, which is critical for the Sam68-PARP1 interaction, is functionally important for PARP1-catalyzed PAR production.

We then performed structural-functional analyses using full-length and truncated PARP1 proteins to explore the domain(s) on PARP1 essential for Sam68 interaction and Sam68-stimulated PARP1 activation. In contrast to the strong interaction between full-length PARP1 and Sam68, PARP1 (663–1,014) truncated protein, containing the catalytic domain, barely bound Sam68 (Fig 4G), which indicates that the stimulatory function of Sam68 on PARP1 activity may not be conferred via a direct interaction between Sam68 and PARP1 catalytic domain. Conversely, the PARP1 (1–662) truncated protein, which contains the DNA-binding domain and automodification domain, associated with Sam68 to a similar extent as full-length PARP1 (Fig 4G), indicative of a potentially important role of the PARP1 N-terminus for the stimulatory function of Sam68. Indeed, in striking contrast to the robust PAR chain formation by Sam68 and PARP1 in the presence of damaged DNA and NAD^+^, incubation of Sam68 with either PARP1 (1–662) or PARP1 (663–1,014) failed to form detectable PAR chains (Fig 4H, compare lanes 18, 20, and 22), suggesting that the Sam68-PARP1 interaction and PARP1 catalytic domain are both required for Sam68 to stimulate PARP1 activation. Moreover, in the absence of damaged DNA, incubation of Sam68 with PARP1 exhibited no detectable PARP1 activation (Fig 4I and S7D Fig), which demonstrates that Sam68 primarily stimulates the DNA-dependent PARP1 activation. Altogether, our results suggest that Sam68 stimulates the DNA-dependent PARP1 activation and subsequent PAR production through the interaction between their N-termini.

### PARP1 Deficiency/Inhibition and Sam68 Deficiency Share Similar Effects on DNA Repair

To recapitulate our observed phenotypes due to diminished PAR synthesis in the absence of Sam68, we performed parallel experiments in PARP1-deficient or -inhibited cells. Indeed, pretreatment of thymocytes with PARP1 inhibitors, Olaparib and PJ-34, significantly impeded γ-irradiation-triggered PAR production and the downstream phosphorylation of ATM and Chk1 (S8 Fig), mirroring the impaired PAR synthesis and DNA repair signaling in Sam68-deficient cells (Fig 2). As a result, PARP1 deletion in MEFs, in comparison to wild-type cells, resulted in increased sensitivity to genotoxicity of etoposide and γ-irradiation (S9A and S9B Fig), which is comparable to Sam68 KO in MEFs (Fig 1A and 1B). Moreover, alkaline comet assays revealed that DNA damage repair was substantially delayed in the PARP1-inhibited thymocytes compared to the control cells, as illustrated by the persistent comet tail moments within 3 h post γ-irradiation (S9C and S9D Fig), further supporting a similar effect of Sam68 deletion and PARP inhibition on DNA repair of γ-irradiated lesions in thymocytes. Furthermore, PARP1 down-regulation by siRNA significantly attenuated the repair efficiency of NHEJ and HDR in the U2OS reporter cell lines, in comparison to the nonspecific control siRNA (S9E Fig), which mirrors the impact of Sam68 silencing on the specific signaling pathways that repair DSBs (Fig 1F). These results suggest that deficiency in either PARP1 or Sam68 similarly causes defective DNA repair, thus resulting in persistent DSBs and hypersensitivity to DNA-damaging agents. To examine whether PARP1 loss further impacts the hypersensitivity of Sam68 KO MEFs to DNA-damaging agents, we down-regulated PARP1 expression by siRNA in Sam68 KO MEFs. Indeed, following exposure to DNA-damaging agents, etoposide and γ-irradiation, the clonogenic survival of Sam68 KO MEFs expressing PARP1-specific siRNA was almost identical to those expressing control siRNA (S10 Fig). These results further highlight the crucial role of Sam68 in controlling the PARP1-catalyzed PAR production in DDR.

### Sam68 Deletion Attenuates PARylation and PAR-Dependent Repair Signaling in Radiodamaged Thymi

**Fig 5:**
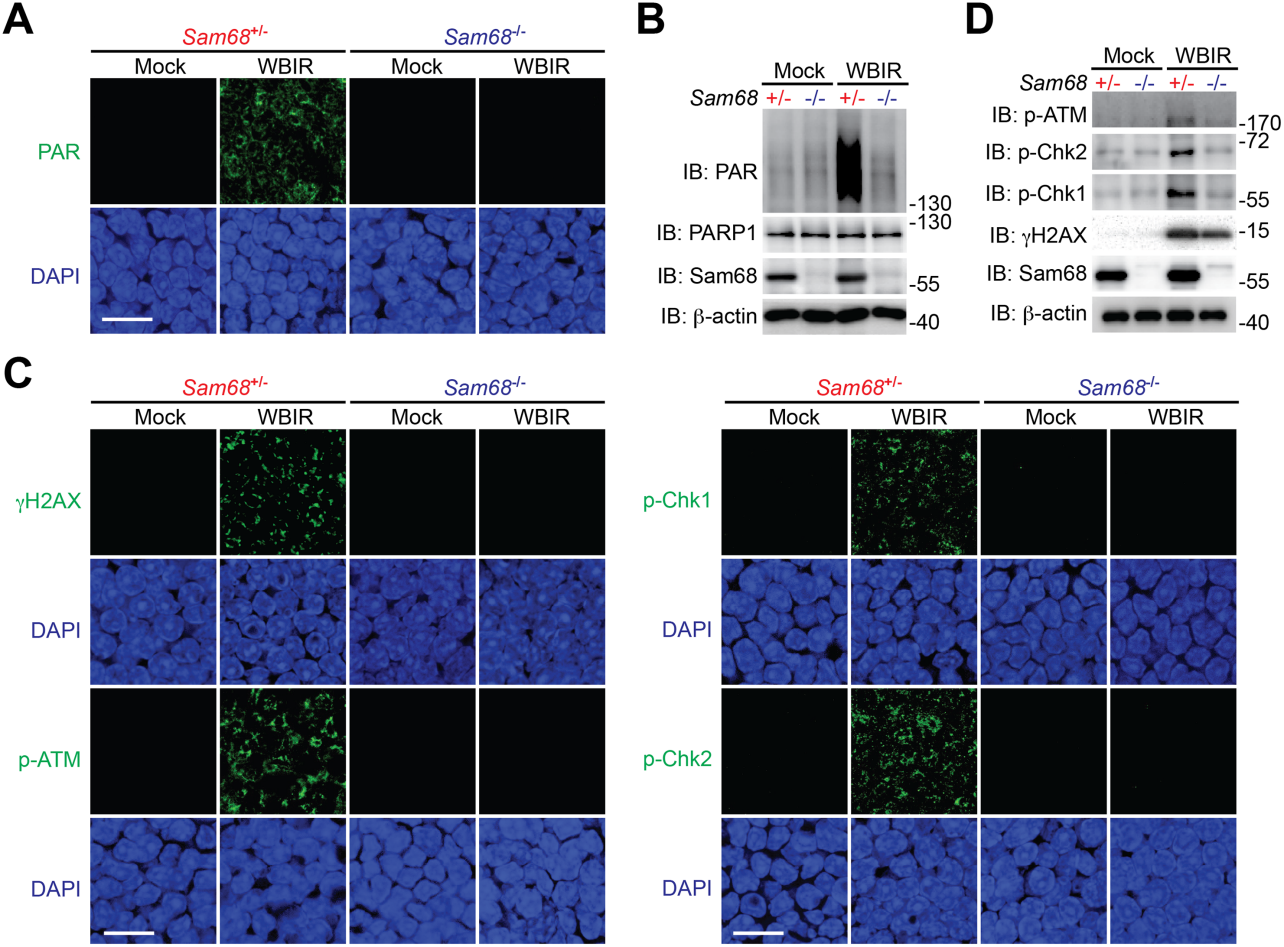
Sam68 deletion dampens DNA repair signaling in the radiodamaged thymus. (A, C) Immunofluorescence micrographs of PAR (A) and phosphorylation of indicated DNA repair signaling molecules (C) in thymi collected from *Sam68*^+/-^ and *Sam68*^-/-^ mice at 20 min following mock-irradiation (Mock) or whole body γ-irradiation (WBIR), with nuclei counterstained by DAPI. Scale bars, 10 μm. (B, D) Whole cell lysates derived from thymocytes collected as in (A, C) were immunoblotted for indicated proteins, with β-actin as a loading control.

In light of the crucial role of Sam68 in mediating DNA repair in cultured cells, we additionally examined the thymi, which is known to be hypersensitive to radiotoxicity [17,63,64], derived from mice at various periods post whole body γ-irradiation (WBIR) to assess the impact of Sam68 on DNA repair signaling in damaged organs in vivo. To assess the immediate effect of Sam68, we harvested the thymi from mock- and γ-irradiated mice at 20 min post WBIR. As visualized by immunohistological staining, the DNA repair signaling in mock-irradiated *Sam68*^+/-^ and *Sam68*^-/-^ mice was comparable (Fig 5A and 5C). Shortly after WBIR, vigorous PAR synthesis and phosphorylation of ATM, Chk1, Chk2, and H2AX were detected in the thymi derived from *Sam68*^+/-^ mice (Fig 5A and 5C). In striking contrast, such a response was greatly diminished in the γ-irradiated *Sam68*^-/-^ thymi (Fig 5A and 5C). Moreover, these results were further supported by IB of thymocyte lysates derived from γ-irradiated *Sam68*^+/-^ and *Sam68*^-/-^ mice (Fig 5B and 5D). Hence, Sam68 is essential for DNA damage-initiated PARylation and repair signaling in radiodamaged thymus.

### Sam68 Deficient Mice Are Hypersensitive to Genotoxic Stresses

**Fig 6:**
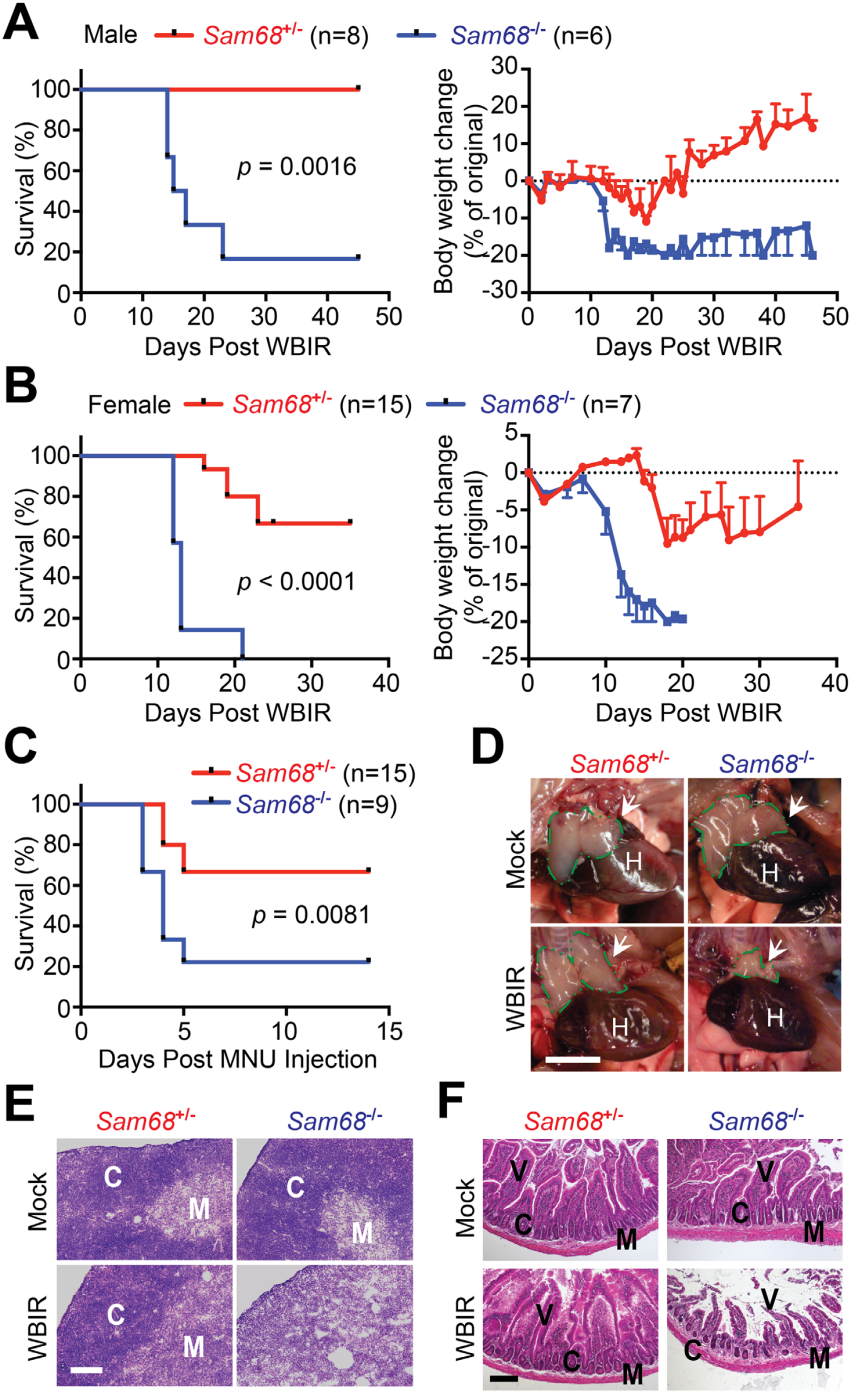
Sam68 KO mice are hypersensitive to genotoxic stresses. (A, B) Kaplan-Meier analysis of the survival rate (left) and normalized body weight changes (right) in male *Sam68*^-/-^ mice and their littermate *Sam68*^+/-^ controls following 6.5 Gy of WBIR (A) and female mice following 7 Gy of WBIR (B). *p* = 0.0016 (A) and *p* < 0.0001 (B), respectively, Gehan-Breslow-Wilcoxon test. (C) Kaplan-Meier analysis of the survival rate in male *Sam68*^+/-^ and *Sam68*^-/-^ mice following N-methyl-N-nitrosourea (MNU) administration at 165 mg/kg of body weight. *p* = 0.0081, Gehan-Breslow-Wilcoxon test. (D) Photographs of thymi derived from *Sam68*^+/-^ and *Sam68*^-/-^ mice at 14 d post mock-irradiation (Mock) or WBIR. Thymi were outlined with dashed green lines and indicated by arrows. H, heart. Scale bar, 5 mm. (E) Hematoxylin and eosin (H&E) staining of thymi as in (D). C, cortex; M, medulla. Scale bar, 100 μm. (F) H&E staining of duodenum from *Sam68*^+/-^ and *Sam68*^-/-^ mice irradiated as in (D). V, villi; C, crypts; M, muscularis externa. Scale bar, 100 μm. Underlying data are shown in S1 Data.

To determine the importance of Sam68 in DDR in vivo, we assessed the impact of Sam68 deletion on γ-irradiation-caused genotoxicity in mice. *Sam68*^+/-^ male mice subjected to a sublethal dose of WBIR initially showed a modest loss in body weight and consistently regained their weight by 18 d post WBIR, correlating with 100% survival from radiotoxicity (Fig 6A). In striking contrast, more severe weight loss and mortality were observed in *Sam68*^-/-^ mice, with a survival rate of 17% over a period of 45 d post WBIR (Fig 6A). Likewise, *Sam68*^-/-^ female mice exhibited hypersensitivity to radiotoxicity compared with their Sam68 sufficient littermates, reflected in a sharp reduction in both body weight and survival rate (Fig 6B). Moreover, we monitored mouse mortality following intraperitoneal administration of N-methyl-N-nitrosourea (MNU), a DNA alkylating agent that causes genotoxicity [17,65]. Over a period of 14 d, *Sam68*^-/-^ mice had higher and accelerated mortality compared to *Sam68*^+/-^ controls (Fig 6C). Together, these results demonstrate a crucial role of Sam68 in protecting mice from genotoxic challenges by γ-irradiation and alkylating chemicals.

We further compared the morphology of the thymus and small intestine in mock- and γ-irradiated animals by gross dissection and histological staining. Of note, the thymic size, structure, and lymphocyte subpopulations of mock-irradiated *Sam68*^+/-^ and *Sam68*^-/-^ mice were comparable, showing that Sam68 deficiency does not impair thymus development (Fig 6D and 6E and S11A Fig). Fourteen d post WBIR, the thymi in *Sam68*^+/-^ mice were indistinguishable to those in mock-irradiated mice in size and morphology (Fig 6D), and all exhibited a well-delineated cortex and medulla zones by histological staining (Fig 6E), indicative of successful DNA repair in the thymi. In contrast, the thymi in γ-irradiated *Sam68*^-/-^ mice were severely damaged, with reduced size and abolished cortex–medulla conjunctions, compared with those from γ-irradiated *Sam68*^+/-^ or mock-irradiated mice (Fig 6D and 6E). Similarly, the small intestine develops normally regardless of Sam68 sufficiency in mice (Fig 6F and S11B Fig). However, *Sam68*^-/-^ mice subjected to WBIR had more widespread damage to the shortened small intestine, particularly to the structure of the villi and crypts in the duodenum (Fig 6F and S11C Fig). The severe damage in the thymus and intestine in *Sam68*^-/-^ mice strongly supports that Sam68 is essential for radioprotection in mice.

## Discussion

**Fig 7:**
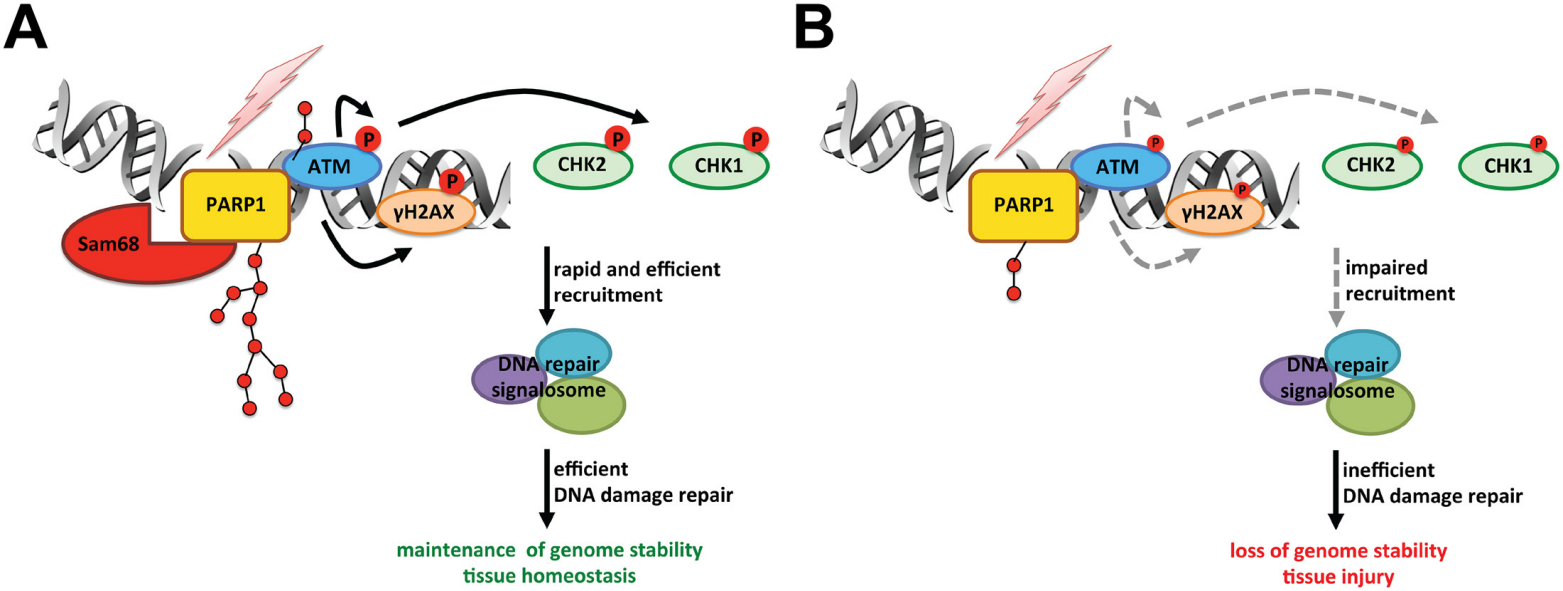
Schematic model representation of Sam68 functioning as a signaling molecule in cellular response to DNA damage. Shown are cellular repair signaling cascades in response to DNA damage in the presence (A) and absence (B) of Sam68.

Herein, we report that Sam68 is a previously unappreciated early signaling molecule in the cellular response to DNA damage. Our data demonstrate that the deletion of Sam68 attenuates DNA damage-triggered PARP1 activation, PAR production, and PAR-dependent DNA repair signaling cascades, in line with our observations of an indispensable role for Sam68 in the HDR and NHEJ repair signaling pathways. Moreover, we show that Sam68 KO mice are hypersensitive to genotoxic challenges by DNA-damaging agents. Mechanistically, upon DNA damage, Sam68 is recruited to DNA damage sites, where Sam68 stimulates the catalytic activity of PARP1 at DNA lesions (Fig 7).

The evidence that Sam68 interacts with PARP1 and is recruited to and substantially overlaps with PARP1 at sites of damaged DNA and that Sam68 deletion diminishes the DSB-initiated PAR production suggests that Sam68 regulates PARP1 activity in DDR. The similarity in the phenotypes of Sam68- and PARP1-deficient/inhibited cells and animals in response to DNA damage further supports this notion. First, dramatically attenuated PAR production in response to DNA damage in Sam68-deficient cells (Fig 2E and 2F) mirrors the abolished DNA damage-triggered PAR synthesis caused by PARP inhibitors (S8 Fig). The role of Sam68 in controlling PARP1 activity is further supported by the evidence that recombinant Sam68 is sufficient to boost PARP1 activity in the presence of damaged DNA (Fig 4A). Secondly, the PAR-dependent DNA repair signaling cascade is dampened in PARP1 KO cells [14,27], PARP1-inhibited cells (S8 Fig), and Sam68-deficient cells (Fig 2A–2D and S3 Fig). Moreover, the retarded repair of DNA strand breaks, as illustrated by comet assays, is similarly observed in PARP1-deficient [66] or -inhibited cells (S9C and S9D Fig) and Sam68-deficient cells (Fig 1C and 1D), in line with the PARP1 malfunction-impeded DNA repair signaling and recruitment of the repair machinery. Hence, Sam68-deleted cells are hypersensitive to genotoxic stress (Fig 1A and 1B), similar to what has previously been reported in cells with repressed PARP1 activity through the use of chemical inhibitors or transdominant mutations [67] and in PARP1-deleted cells (S9A and S9B Fig). Furthermore, owing to its key function in DDR, PARP1 is known to be required for protecting mice from genotoxicity. PARP1 KO mice were reported previously to be hypersensitive to the DNA alkylating agent MNU and γ-irradiation [17,65], and γ-irradiation has been shown to cause severe acute damage to the small intestine in PARP1 KO mice [17]. Interestingly, Sam68 KO mice are also hypersensitive to MNU and γ-irradiation challenges and exhibit acute radiodamage in the small intestine and the thymus (Fig 6), thus exactly recapitulating the phenotypes in PARP1 KO mice.

One hallmark of DDR factors is that they are rapidly recruited to the proximity of sites of damaged DNA, and ideally, such enrichment can be visualized by laser microirradiation microscopy [1,68–70]. It has been widely acknowledged that activated PARP1 PARylates itself with such automodification stimulating more PARP1 to be recruited to DNA damage sites (Fig 3F) [2]. Notably, in our laser microirradiation microscopy assays, the accumulation of ectopically expressed and endogenous Sam68 also leads to the formation of discrete, cytologically detectable foci at sites of damaged DNA that significantly overlap with the damage foci formed by GFP-PARP1 or endogenous γH2AX (Fig 3F and S6A and S6B Fig). In addition, we demonstrate that Sam68 and PARP1 are specifically enriched on the chromatin close to the unique DSB at the I-SceI site (Fig 3E). Moreover, Sam68 and PARP1 are capable of physically interacting with each other (Fig 4C), and DNA damage-enhanced interactions between endogenous Sam68 and key initial components of DDR, i.e., PARP1 and ATM (Fig 3A and S4 Fig). Furthermore, recombinant Sam68 is sufficient to facilitate the DNA-dependent PARP1 activity in vitro (Fig 4A and S7D Fig), and the N-terminus of Sam68 is both critical for the Sam68-PARP1 interaction and functionally important for PARP1-catalyzed PAR production (Fig 4B–4D). Together, these results suggest that Sam68, functioning as a key initial signaling molecule, is crucial in regulating PARP1 activity and the PAR-dependent signaling cascade in DDR. It is noteworthy that our work here also elucidates a novel role for the less-characterized N-terminus of Sam68 in DDR signaling. Albeit not appearing to harbor any well-defined functional domains or motifs, the N-terminus of Sam68 does contain a cluster of several serine and threonine residues. This cluster could make Sam68 a potential target of serine/threonine phosphorylation, thus potentially serving as an important functional switch that allows Sam68 to interact with PARP1 and regulate PARP1-catalyzed PARylation in DDR, though further studies are needed to confirm this hypothesis.

Recent studies underscore the emerging role of RNA-binding proteins in DDR [52–54]. Heterogeneous nuclear ribonucleoprotein U-like proteins 1 and 2 were shown to promote recruitment of the Bloom Syndrome protein (BLM) helicase to DSBs by functioning downstream of Mre11-Rad50-Nbs1 (MRN) complex and carboxyl-terminal binding protein (CtBP)-interacting protein [53]. Moreover, RNA-binding proteins RNA binding motif protein, X-linked (RBMX) and non-POU domain containing, octamer-binding (NONO) were reported to be recruited to DNA damage sites in a PARP1/PAR-dependent manner, where RBMX regulates BRCA2 expression [54] and NONO stimulates NHEJ and represses HR repair signaling pathways [52], respectively. It is noteworthy that all these RNA-binding proteins have been proposed to function after PARP1 activation/PAR synthesis in DDR, as their enrichment on DNA lesions relies on the interactions with PAR chains [52]. In contrast, here we propose that the RNA-binding protein Sam68 is a signaling molecule that functions prior to PARP1 activation and in fact controls PARP1 activity in DDR (Fig 7). The enrichment of Sam68 on DNA lesions is apparently as rapid as PARP1, and it stimulates and controls rather than depends on PARP1 activation and PAR production. Thus, the identification of Sam68 as an early signaling factor proximal to PARP1 in DDR provides new insight into the sophisticated signaling mechanisms in DDR, in particular the stimulation and regulation of PARP1 activation and PAR production.

In light of their crucial roles in various DNA damage repair-signaling pathways, the inhibition of PARP1 as well as other PARP family proteins has emerged as a promising therapeutic approach for treating multiple human diseases associated with impaired DNA repair activities [34,35]. However, the current classes of PARP inhibitors undergoing clinical trials and the FDA-approved Olaparib are all based on a competitive binding strategy first observed with nicotinamide [36], and almost all PARP inhibitors are derivatives of the natural PARP inhibitor nicotinamide [10]. There is an urgent need for improving their specificity for each individual PARP family member and lowering their off-target effect and toxicity [2]. That said, the nature of PARP1’s rapid recruitment (within seconds) to DNA damage sites upon sensing lesions combined with the fact that activated PARP1 vigorously catalyzes the PARylation reaction makes it difficult to elucidate the mechanism of PARP1 activation in DDR. Nevertheless, PARP activation and PAR production need to be elegantly controlled during the cellular response to DNA damage—a sufficient amount of PAR at DNA damage sites is required to recruit downstream effectors to DNA lesions to fulfill their repair function. Meanwhile, excessive PAR chain buildup needs to be avoided, as hyperactivated PARP1 could exhaust intracellular pools of NAD^+^ and sabotage ATP production, thus resulting in cell death [71–74]. Although post-translational modifications and interactions with other proteins have been proposed to fine-tune PARP1 activity in DDR [32,75], the stimulatory mechanism of PARP1 activation at DNA damage sites has remained largely unknown. Our proposed model that Sam68, as a previously unrecognized stimulatory factor beyond DNA strand breaks, stimulates PARP1 activation and PAR production at DNA damage sites could provide a novel strategy to develop a new category of PARP1 inhibitors and therapeutics for human diseases involving DNA repair.

## Materials and Methods

### Mice

All animal experiments were performed according to protocol number MO13-H349, approved by the Johns Hopkins University’s Animal Care and Use Committee and in direct accordance with the NIH guidelines for housing and care of laboratory animals. *Sam68*^-/−^ (Sam68 KO) mice and their gender-matched littermate *Sam68*^+/−^ heterozygous mice (occasionally substituted with gender-matched littermate *Sam68*^+/+^ mice when *Sam68*^+/−^ ones were lacking but referred to as *Sam68*^+/−^ alone for simplicity) were produced using heterozygous breeding pairs and were genotyped for disrupted or wild-type *Sam68* gene, as previously described [39]. Mice were maintained in a specific pathogen-free facility and fed autoclaved food and water ad libitum.

### Cell Culture, Reagents, and Plasmids

Wild-type, Sam68 KO, and PARP1 KO MEFs were kindly shared by Drs. Stephan Richard (McGill University, Canada) and Zhao-Qi Wang (Fritz Lipmann Institute, Germany). Wild-type U2OS cells and the DR-GFP and EJ5-GFP reporter U2OS cells [58] were kindly provided by Drs. Michael Matunis (Johns Hopkins University) and Jeremy Stark (Beckman Research Institute of City of Hope). MEF and U2OS cells were cultured in DMEM medium containing 10% fetal calf serum, 2 μM glutamine, and 100 U/ml each of penicillin and streptomycin, except for the addition of 10 mM HEPES (pH 7.2–7.5) for U2OS culture. U2OS reporter cells were cultured using similar medium as for U2OS cells, except without sodium pyruvate. Antibodies used were as follows: Sam68, GST, PARP2, PARP3, and PARP5a/b from Santa Cruz Biotechnology (Dallas, Texas); β-actin and myc from Sigma-Aldrich (St. Louis, Missouri); ATM, PARP1, Caspase-3, Chk1, Chk2, p-Chk1 (Ser345), p-Histone H3 (Ser10), and β-Catenin from Cell Signaling Technology (Danvers, Massachusetts); PAR from Trevigen (Gaithersburg, Maryland); γH2AX from Millipore (Billerica, Massachusetts); H2AX from Bethyl Laboratories (Montgomery, Texas); p-ATM from Rockland (Gilbertsville, Pennsylvania); p-Chk2 from Novus Biologicals (Littleton, Colorado); RFP from GenScript (Piscataway, New Jersey); Sam68 from GeneTex (Irvine, California); Histone3 from Abcam (Cambridge, Massachusetts); CD4 and CD8α from BioLegend (San Diego, California). MNU, etoposide (VP16), 4′, 6-diamidino-2-phenylindole (DAPI), and ethidium bromide (EtBr) were obtained from Sigma-Aldrich. 4-[(3-[(4-cyclopropylcarbonyl)piperazin-4-yl]carbonyl)-4-fluorophenyl]methyl(2H)phthalazin-1-one (Olaparib) and *N*-(6-oxo-5,6-dihydrophenanthridin-2-yl)-*N*, *N*-dimethylacetamide-HCl (PJ-34) were purchased from Fisher Scientific (Pittsburgh, Pennsylvania) and Enzo Life Sciences (Farmingdale, New York), respectively. The GFP-PARP1, RFP-Sam68, and I-SceI plasmids were kindly shared by Drs. Anthony Leung (Johns Hopkins University), Johnny He (University of North Texas Health Science Center), and Jeremy Stark (Beckman Research Institute of City of Hope), respectively. The GFP, GFP-Sam68, GFP-Sam68 (ΔC), GFP-Sam68 (ΔN), GFP-Sam68 (ΔKH), GST, GST-Sam68, and GST-Sam68 (ΔN) constructs were described previously [51].

### γ-Irradiation

The γ-irradiation on MEFs, U2OS cells, and primary thymocytes was performed using a ^137^Caesium source (dose rate 4 Gy/min). WBIR in mice was performed as previously described [17,65]. Briefly, 6–8-wk-old *Sam68*^+/-^ and *Sam68*^-/-^ mice were subjected to a single dose of sublethal γ-irradiation from an MSD Nordion Gammacell 40 Exactor, with a dual ^137^Caesium source (dose rate 1 Gy/min). The body weight, mortality, and survival of mice were monitored post irradiation, and in some circumstances, the γ-irradiated mice were sacrificed at indicated time points post WBIR for histological and immunohistological analyses.

### Proliferation Assays

The in vitro proliferation assays were performed as previously described [76]. Briefly, 1 × 10^3^ MEF cells were γ-irradiated at the indicated dose and were seeded in the wells of a 6-well plate immediately after γ-irradiation. In certain cases, 1 × 10^3^ MEF cells seeded in DMEM medium were treated with or without indicated concentrations of etoposide for 20 h, followed by extensive washes with phosphate-buffered saline (PBS). After incubation for an additional 96 h, the surviving cells were accounted using a Z1 Coulter Particle Counter (Beckman Coulter, Indianapolis, Indiana), and the survival fractions were calculated by comparing the live cell numbers in treated cultures to those in untreated controls.

### Alkaline Comet Assays

Comet assays were conducted by using the Comet Assay Kit (Trevigen) following the manufacturer’s instructions. Briefly, isolated primary mouse thymocytes were mock-treated or γ-irradiated with indicated doses and then allowed to recover in normal DMEM culture medium containing 10 mM HEPES (pH 7.2–7.5) for indicated periods at 37°C. Cells were collected and washed once with PBS, and 3 × 10^5^ cells were combined with 1% molten LMAgarose at 37°C at a ratio of 1:10 (v/v) and immediately pipetted onto slides. Slides were then immersed in prechilled lysis solution for 1 h on ice to lyse cells, followed by alkaline unwinding of chromatin. Alkaline electrophoresis of gelled slides was performed using an Econo-Sub Horizontal System (C.B.S. Scientific, Del Mar, California) at 24V (0.7 V/cm) at 4°C for 30 min. The DNA was visualized by SYBR Green staining, and images were taken under an Axio Observer fluorescence microscope (Zeiss, Oberkochen, Germany) and analyzed by CometScore software (TriTek, Sumerduck, Virginia).

### RNA Interference and Transfection

The siRNAs targeting human Sam68 were described previously [51]. Human and mouse PARP1 siRNAs were purchased from Santa Cruz Biotechnology. Transient transfection of siRNA or plasmids cells was performed using Lipofectamine RNAiMax and Lipofectamine 2000 (Life Technologies, Frederick, Maryland), respectively, according to the manufacturer's instructions.

### DNA Double-Strand Break Repair Reporter Assays

DSB repair assays were conducted as previously described [58]. In brief, 6 × 10^5^ U2OS cells (DR-GFP/homologous-directed repair and EJ5-GFP/NHEJ) were transfected with nonspecific control or Sam68-specific siRNAs. Forty-four hours later, cells were transfected again with siRNAs together with I-SceI (to generate a DSB at the unique I-SceI site) or GFP (to indicate transfection efficiency) plasmids. Seventy-two hours later, cells were collected and subjected to flow cytometry analysis for GFP positive cells.

### Flow Cytometry

For flow cytometry, 0.5–2 × 10^6^ cells were washed twice with PBS, resuspended in staining buffer (1% fetal bovine serum in PBS), and stained with appropriate antibodies for cell surface markers on ice for 30 min. Following staining and extensive washes with staining buffer, cells were analyzed on a FACSCalibur (BD Biosciences, San Jose, California). Events were collected and analyzed with the FlowJo software (Tree Star, Ashland, Oregon).

### Isolation of Primary Thymocytes

Freshly excised thymi from mice were gently teased with a syringe and forceps in DMEM containing 10 mM HEPES (pH 7.2–7.5). The mechanically disrupted cell clumps were poured through 70 μm nylon mesh cell strainers (BD Falcon, Bedford, Massachusetts) to remove connective tissue and prepare single cell suspensions. The cell suspensions were washed once with PBS, and the red blood cells were lysed with Ammonium-Chloride-Potassium (ACK) buffer. The remaining thymocytes were counted, washed twice in PBS, and recovered in DMEM containing 10% fetal calf serum, 2 μM glutamine, 100 U/ml each of penicillin and streptomycin, and 10 mM HEPES (pH 7.2–7.5) for 1 h, followed by further treatments as indicated.

### Immunoprecipitation and Immunoblot

Immunoprecipitation and immunoblot assays were conducted as previously described [51]. In brief, the cells were harvested and lysed on ice by 0.4 ml of lysis buffer (50 mM Tris-HCl [pH 8.0], 150 mM NaCl, 1% NP-40 and 0.5% sodium deoxycholate, 1 × complete protease inhibitor cocktail [Roche Applied Science, Indianapolis, Indiana]) for 30 min. The lysates were centrifuged at 10,000 × *g* at 4°C for 10 min. The protein-normalized lysates were subjected to immunoprecipitation by adding 10 mg/ml of the appropriate antibody, 30 μl of protein G-agarose (Roche Applied Science), and rotating for more than 2 h at 4°C. The precipitates were washed at least four times with cold lysis buffer followed by a separation by SDS-PAGE under reduced and denaturing conditions. The resolved protein bands were transferred onto nitrocellulose membranes, probed as described previously [77,78], developed by the Super Signaling system (Thermo Scientific, Waltham, Massachusetts) according to the manufacturer's instructions, and imaged using a FluorChem E System (Protein Simple, Santa Clara, California).

### Immunofluorescence Microscopy

Immunofluorescence microscopy was performed as previously described [51,79]. Briefly, cells were fixed with 4% paraformaldehyde in PBS and then Cellspin mounted onto slides. After a 5-min permeabilization with 0.05% Triton X-100 in PBS and a 30-min blocking with 5% goat serum, the fixed cells were stained with the appropriate primary antibodies for 1 h and with fluorescence dye-conjugated second antibodies (Life Technologies) for 1 h together with 1 μg/ml of DAPI (Sigma-Aldrich) for 5 min at 25°C. The slides were then rinsed with PBS three times and cover mounted for fluorescence microscopy.

### Chromatin Fractionation

Cells were harvested at indicated time points after γ-irradiation, and cell pellets were resuspended in the NETN buffer (20mM Tris–HCl [pH 8.0], 100 mM NaCl, 1mM EDTA, and 0.5% NP-40) and incubated on ice for 20 min. Supernatant after 3,000 × *g* for 10 min was collected as soluble fraction. Pellets were recovered and resuspended in 0.2 M HCl on ice for 30 min and sonicated for 10 s to release chromatin-bound proteins; then, the soluble fractions were neutralized with 1 M Tris–HCl (pH 8.5) and collected as chromatin fraction, and the pellets were collected as insoluble fraction for further analysis, as described previously [33,76].

### ChIP

ChIP assays were performed as previously described [79]. Briefly, U2OS DR-GFP cells were transfected with a plasmid encoding I-Scel endonuclease to create a DSB. At 20 h post transfection, 4.5 × 10^6^ cells were fixed by 1% formaldehyde at room temperature for 10 min, and the cross-linking reaction was stopped by 125 mM of glycine. Cells were homogenized in cell lysis buffer (5 mM PIPES, 85 mM KCl, 0.5% NP-40, 1 × protease inhibitor cocktail [Roche]), and the nuclei were resuspended in nuclei lysis buffer (50 mM Tris-HCl [pH 8.1], 10 mM EDTA, 1% SDS, 1 × protease inhibitor cocktail) on ice for 10 min and sonicated by Bioruptor UCD-200 (Life Technologies). The extract was then clarified by centrifugation and diluted 10-fold with dilution buffer (16.7 mM Tris [pH 8.1], 167 mM NaCl, 1.2 mM EDTA, 1.1% Triton X-100, 0.01% SDS) to yield the solubilized chromatin. For immunoprecipitation, anti-Sam68, anti-PARP1, or IgG control antibody, and Protein AG magnetic beads (Thermo Scientific) were added to the soluble chromatin, and the mixture was incubated at 4°C overnight. The beads were then washed sequentially with TSE-150 mM NaCl (20 mM Tris [pH 8.1], 2 mM EDTA, 1% Triton X-100, 0.1% SDS, and 150 mM NaCl), TSE-500 mM NaCl, buffer III (10 mM Tris [pH 8.1], 0.25 M LiCl, 1% NP-40, 1% deoxycholate, 1 mM EDTA) and three times with TE (10 mM Tris 8, 1 mM EDTA). The immunoprecipitants were eluted by elution buffer (1% SDS, 50 mM NaHCO_3_, 20 μg/ml glycogen), and DNA was extracted with phenol-chloroform and resuspended in TE. PCR (30–35 cycles) was performed using MyTaq Red Mix (Bioline, Taunton, Massachusetts) with following primers at or adjacent to the unique I-SceI site: P1F, 5′-GAGCAAGGGCGAGGAGCTGT-3′; P1R, 5′-CCGTAGGTCAGGGTGGTCAC-3′; P2F, 5′-TCTTCTTCAAGGACGACGGCAACT-3′; P2R, 5′-TGCCGTTCTTCTGCTTGTC-3′; P3F, 5′-CCGCGACGTCTGTCGAGAAG-3′; and P3R, 5′-GCCGATGCAAAGTGCCGATA-3′.

### GST Pulldown Assays

The GST pulldown assays were performed as previously described [79]. Briefly, 1 μg of GST/Ni^2+^ affinity-purified recombinant protein, as indicated, was applied to glutathione Sepharose 4B resins (Amersham Pharmacia) and incubated for 1 h at 4°C. After washing, bound proteins were eluted and subjected to SDS-PAGE, followed by immunoblotting.

### Laser Microirradiation Microscopy

Laser microirradiation microscopy assays were performed as previously described [62,80], with some modifications. Briefly, near infrared (NIR) excitation was provided by a Mai Tai HP Ti:Sapphire laser (Spectra Physics, Santa Clara, California) tuned to 800 nm, 125 fs pulses, and an 80 MHz repetition rate. The laser beam was introduced through a Leica DMi8 confocal microscope (Leica Microsystems, Mannheim, Germany). The pulsed NIR beam was focused to a diffraction-limited spot with a 63 × oil-immersion objective (1.4 NA). An electro-optic modulator (EOM) was used to create regions of interest (ROIs) targeted for microirradiation by position-dependent power adjustment of the NIR beam. Prior to every experiment, the NIR beam power at the objective was measured to ensure repeatable laser settings that generated a detectable DDR restricted to ROIs without noticeable cytotoxicity. A coverglass-bottomed dish (MatTek Corporation, Ashland, Massachusetts) plated with cells was placed into a temperature-controlled chamber (37°C, 5% CO_2_) (Life Imaging Services, Basel, Switzerland) enclosing the entire microscope stand. Immediately after microirradiation, the DDR was captured through time-lapse acquisition of the GFP- or RFP-fused protein recruitment using the FRAP application unit of LAS X acquisition software. All images were processed using the LAS AF Lite software (Leica Microsystems), and we quantified the pre- and post-microirradiation fluorescence intensity of the ROI and an adjacent nuclear area with a similar pre-microirradiation fluorescence intensity, to correct fluorescence intensity for transfection efficiency and photobleaching.

### MNU Administration

MNU injection-induced genotoxic stress in mice was carried out as previously described [17,65]. Briefly, 6–8-wk-old *Sam68*^+/-^ and *Sam68*^-/-^ mice were administrated a single dose of 165 mg/kg body weight of MNU in 200 μl of PBS by intraperitoneal injection. The body weight, mortality, and survival of mice were monitored until 14 d post injection.

### Histology

After euthanizing mice, the thymus was removed, washed once with ice-cold PBS, immersed in optimal cutting temperature media (Tissue-Tek, Elkhart, Indiana), and frozen in dry ice to preserve the tissue. The duodenum and colon were removed, washed once with ice-cold PBS, fixed in 4% PFA at room temperature for 24 h, and embedded in paraffin. Five-micron sections were cut for all tissues and processed for hematoxylin and eosin (H&E) staining. Stained sections were microphotographed to perform histomorphometric analyses, as previously described [81].

### Immunohistology

Immunohistology was carried out as previously described [81]. In Brief, after euthanizing mice, the thymus was excised under aseptic conditions and frozen in optimal cutting temperature media (Tissue-Tek). Five-micron frozen sections were cut using a Microm HM 550 Cryostat (Thermo Scientific), collected on coated slides, fixed in 4% paraformaldehyde, washed with PBS, and blocked with appropriate sera in PBS. After incubating with appropriate antibodies, sections were washed and incubated with fluorescence dye-conjugated second antibodies and 1 μg/ml of DAPI (Sigma). Stained sections were washed and mounted under a coverslip using Fluoro-gel with Tris Buffer (Electron Microscopy Sciences, Hatfield, Pennsylvania) and examined using an Axio Observer fluorescence microscope (Zeiss).

### In Vitro PARylation Assays

For in vitro PARylation assays, PARP1, PARP1 (1–662), PARP1 (663–1,014), GST, GST-Sam68, and GST-Sam68 (ΔN) recombinant proteins were purified as previously described [82] using three chromatographic steps: GST/Ni^2+^ affinity, heparin-sepharose, and gel filtration. In vitro PARylation assays were performed as previously described [83]. Briefly, the indicated recombinant proteins were incubated for 20 min at 30°C in a standard assay buffer (100 mM Tris-HCl [pH 8.0], 10 mM MgCl_2_, 10% (v/v) glycerol, and 1.5 mM DTT) in the presence and absence of damaged DNA (sonicated) and NAD^+^. The reaction was terminated by the addition of SDS sample buffer (Life Technologies), and the boiled samples were subjected to SDS-PAGE. When indicated, the PARP inhibitor PJ-34 was added to the reaction mixture at a final concentration of 1 μM for 15 min prior to the reaction.

### Statistical Analysis

All statistical analysis was performed using GraphPad Prism version 6.0 (GraphPad Software, San Diego, California). The differences between treated and control groups were examined by unpaired Student’s *t* tests, except Gehan-Breslow-Wilcoxon tests were used for Kaplan-Meier survival curves. Standard errors of means (SEMS) were plotted in graphs. ns means nonsignificant difference, and significant differences were considered * at *p* < 0.05; ** at *p* < 0.01; *** at *p* < 0.001; and **** at *p* < 0.0001.

## Supporting Information

S1 Data Excel spreadsheet containing, in separate sheets, the underlying numerical data and statistical analysis for the following figure panels: Figs 1A, 1B, 1D, 1F, 6A–6C and S1, S2, S6C–S6E, S7B, S9A, S9B, S9D, S9E, S10A and S10B Figs. (XLSX)

S1 Fig Sam68 is required for clonogenic survival of MEFs following exposure to H_2_O_2_. Survival fraction of wild-type (WT) and Sam68 KO MEFs 96 h post treatment with indicated concentrations of H_2_O_2_ for 15 min. Results are expressed as mean and SEM. ***, *p* < 0.001; ****, *p* < 0.0001 by Student’s *t* tests. Underlying data are shown in S1 Data.
(TIF)

S2 Fig Sam68 is critical for repairing DNA damage in U2OS reporter cell lines. U2OS reporter cell lines, specifically designed to repair DNA damage through NHEJ and HDR, were transfected with nonspecific control (si-NC) or Sam68-specific (si-Sam68) siRNA. Forty-eight hours later, cells were transfected with siRNAs and RFP or siRNA-resistant RFP-Sam68, as indicated, together with (+) or without (−) I-SceI plasmid. Another 72 h later, cells were harvested for flow cytometric analyses of the DNA damage repair efficiency in the indicated reporter cell lines. The relative repair efficiency (normalized to si-NC, RFP, and I-SceI cotransfected cells) was quantified from three independent experiments. Results are expressed as mean and SEM. ns, nonsignificant difference; *, *p* < 0.05; **, *p* < 0.01; by Student’s *t* tests. Underlying data are shown in S1 Data.
(TIF)

S3 Fig Sam68 deficiency attenuates DNA repair signaling in cell culture. (A, B) U2OS cells transiently transfected with si-NC or si-Sam68 siRNA (A) or WT and Sam68 KO MEFs (B) were treated with 4 Gy of γ-irradiation (IR). Whole cell lysates were derived at indicated time points following IR and immunoblotted for indicated proteins, with β-actin as a loading control.
(TIF)

S4 Fig Sam68 interacts with early DNA damage signaling molecules PARP1 and ATM. (A) Coimmunoprecipitation showing the inducible Sam68-PARP1 interaction. WT MEFs were γ-irradiated at 10 Gy, and whole cell lysates (Input) derived at indicated time points post irradiation were immunoblotted directly or after immunoprecipitated (immunoprecipitation, IP) with PARP1 or isotype control antibody for indicated proteins. (B) WT and Sam68 KO MEFs were γ-irradiated as in (A). Whole cell lysates (Input) derived at the indicated periods post IR were immunoblotted directly or after immunoprecipitated with Sam68 antibody for the indicated proteins.
(TIF)

S5 Fig Chromatin fractionation assays showing dynamics of Sam68 on damaged chromatin. U2OS cells were γ-irradiated at 10 Gy, and the chromatin, soluble (Sol. fr.), and insoluble (Ins. fr.) subcellular fractions were derived at indicated time points following IR and immunoblotted for indicated proteins. Casp-3, Caspase-3; H3, Histone H3.
(TIF)

S6 Fig Sam68 localizes to sites of DNA damage in DDR. (A) Sam68 KO MEFs transiently expressing RFP-Sam68 together with GFP-PARP1 were subjected to laser microirradiation (Micro-IR). Cells were fixed at 1 min post Micro-IR and stained for endogenous γH2AX. Shown are fluorescence micrographs of RFP-Sam68, GFP-PARP1, and endogenous γH2AX at DNA damage foci. (B) Immunofluorescence micrographs of endogenous Sam68, PARP1, and γH2AX in WT MEFs at 1 min post Micro-IR. (C) WT and Sam68 KO MEFs expressing GFP-PARP1 were subjected to Micro-IR and the increase in relative fluorescence intensity (RFI) of GFP-PARP1 at damage foci ~10 s post Micro-IR versus pre-Micro-IR in WT, and Sam68 KO MEFs were graphed, normalized to WT controls. (D) WT MEFs expressing GFP-PARP1 were pretreated with DMSO or 10 μM of Olaparib for 90 min, followed by Micro-IR. The increase in RFI of GFP-PARP1 at damage foci ~10 s post Micro-IR versus pre-Micro-IR in DMSO- or Olaparib-treated cells was graphed, normalized to DMSO controls. (E) Sam68 KO MEFs expressing RFP-Sam68 were pretreated with DMSO or 10 μM of Olaparib for 90 min, followed by Micro-IR. The increase in RFI of RFP-Sam68 at damage foci ~10 s post Micro-IR versus pre-Micro-IR in DMSO- or Olaparib-treated cells was graphed, normalized to DMSO controls. Results in (C–E) are expressed as mean and SEM. ns, nonsignificant difference; *, *p* < 0.05 by Student’s *t* tests. Underlying data are shown in S1 Data.
(TIF)

S7 Fig The DNA-dependent PARP1-mediated PARylation in vitro. (A) Recombinant PARP1 protein was incubated in reaction buffer in the presence and absence of damaged DNA, NAD^+^, and PARP inhibitor PJ-34, as indicated. The reaction mixture was separated by SDS-PAGE and subjected to immunoblotting with the PAR antibody. (B) GST control or increasing amount of GST-Sam68 recombinant proteins were incubated with recombinant PARP1 protein in reaction buffer containing damaged DNA and NAD^+^, with indicated final concentrations. The reaction mixture was separated by SDS-PAGE and subjected to IB with the PAR and GST antibodies. Right, quantification of relative PARP1 activity based on PAR band density (normalized to the GST control), summarized from three independent experiments. Results are expressed as mean and SEM. *, *p* < 0.05 by Student’s *t* tests. (C) The indicated recombinant proteins were incubated in reaction buffer containing damaged DNA and NAD^+^. The reaction mixture was separated by SDS-PAGE and subjected to IB with the PAR antibody. (D) The indicated recombinant proteins were incubated in reaction buffer containing NAD^+^ in the presence and absence of damaged DNA. The reaction mixture was separated by SDS-PAGE and subjected to IB with the PAR and GST antibodies. Underlying data are shown in S1 Data.
(TIF)

S8 Fig PARP inhibition attenuates DNA damage-induced repair signaling in thymocytes. (A, B) *Sam68*^+/-^ thymocytes pretreated with Olaparib (20 μM) (A), PJ-34 (20 μM) (B), or DMSO for 2 h were γ-irradiated at 4 Gy, and whole cell lysates were derived at indicated time points and immunoblotted for the indicated proteins, with β-actin as a loading control.
(TIF)

S9 Fig PARP1 is critical for repairing DNA strand breaks. (A, B) Survival fraction of WT and PARP1 KO MEFs 96 h post treatment with indicated concentrations of etoposide for 20 h (A) or indicated doses of IR (B). (C) WT thymocytes were pretreated with DMSO or 20 uM of Olaparib, followed by 4 Gy of IR or mock-irradiation. Cells were harvested at the indicated time points post IR and subjected to alkali comet assay. Shown are representative microphotographs. (D) Quantification of tail moments in (C), with summarized data from 40–60 cells within 15 random fields for each time point. (E) U2OS reporter cell lines, specifically designed to repair DNA damage through NHEJ and HDR, were transfected with si-NC or PARP1-specific (si-PARP1) siRNA, together with (+) or without (−) I-SceI plasmid, or GFP control. Seventy-two hours later, cells were harvested for flow cytometric analyses of the effect of PARP1 knockdown on DNA damage repair efficiency in the indicated reporter cell lines. The relative repair efficiency (normalized to si-NC and I-SceI cotransfected cells) was quantified from three independent experiments. The PARP1 knockdown efficiency was examined by immunoblotting, with β-actin as a loading control, in the indicated reporter cell lines (bottom). Results in A–B and D–E are expressed as mean and SEM. ns, nonsignificant difference; *, *p* < 0.05; **, *p* < 0.01; ***, *p* < 0.001; ****, *p* < 0.0001 by Student’s *t* tests. Data are representative of two independent experiments. Underlying data are shown in S1 Data.
(TIF)

S10 Fig PARP1 knockdown does not impact the sensitivity of Sam68-deleted cells in response to DNA damage. (A, B) Survival fraction of Sam68 KO MEFs silenced with si-NC or si-PARP1 siRNA 96 h post treatment with indicated concentrations of etoposide for 20 h (A) or indicated doses of IR (B). (C) The PARP1 knockdown efficiency was examined by immunoblot, with β-actin as a loading control, in Sam68 KO MEFs at 96 h after siRNA transfection. Underlying data are shown in S1 Data.
(TIF)

S11 Fig Sam68 deficiency does not affect thymus and small intestine development in mice but causes more severe damage post IR. (A) Representative flow cytometry analysis of indicated immune cell subpopulations in thymocytes derived from naïve *Sam68*^+/-^ and *Sam68*^-/-^ mice. (B, C) Photographs of small intestines collected from *Sam68*^+/-^ and *Sam68*^-/-^ mice at 14 d post mock-irradiation (B) or WBIR (C). Scale bars, 2 cm.
(TIF)

## Abbreviations

ACK: Ammonium-Chloride-Potassium
ARTD: diphtheria toxin-like ADP-ribosyl transferase
ATM: ataxia telangiectasia mutated
ATR: ataxia telangiectasia and Rad3-related
BER: base excision repair
BLM: Bloom Syndrome protein
Casp-3: Caspase-3
CtBP: carboxyl-terminal binding protein
Chk1: checkpoint kinase 1
Chk2: checkpoint kinase 2
ChIP: chromatin immunoprecipitation
DAPI: 4′, 6-diamidino-2-phenylindole
DDR: DNA damage response
DNA-PK: DNA-dependent protein kinase
DSB: DNA double-strand break
EOM: electro-optic modulator
EtBr: ethidium bromide
GFP: green fluorescent protein
H3: Histone H3
HDR: homology-directed repair
H&E: hematoxylin and eosin
HR: homologous recombination
IB: immunoblot(ting)
IP: immunoprecipitation
KO: knockout
MEF: mouse embryonic fibroblast
MNU: N-methyl-N-nitrosourea
MRN: Mre11-Rad50-Nbs1
NAD^+^: nicotinamide adenine dinucleotide
NHEJ: nonhomologous end joining
NIR: near infrared
NONO: non-POU domain containing, octamer-binding
PAR: polymers of ADP-ribose
PARP1: poly(ADP-ribose) polymerase 1
PARylation: poly(ADP-ribosyl)ation
PBS: phosphate-buffered saline
RBMX: RNA binding motif protein, X-linked
RFI: relative fluorescence intensity
RFP: red fluorescent protein
ROI: region of interest
Sam68: Src-associated substrate during mitosis of 68 kDa
SEM: standard error of the mean
si-NC: nonspecific control siRNA
si-PARP1: PARP1-specific siRNA
si-SAM68: Sam68-specific siRNA
siRNA: small interference RNA
SSBR: single-strand break repair
WBIR: whole body γ-irradiation
WT: wild type
