# Supplementary figures and images for: Sam68 Is Required for DNA Damage Responses via Regulating Poly(ADP-ribosyl)ation
Source: PLoS Biol. 2016 Sep 16;14(9):e1002543. doi: 10.1371/journal.pbio.1002543 (PMC5026359; doi:10.1371/journal.pbio.1002543)

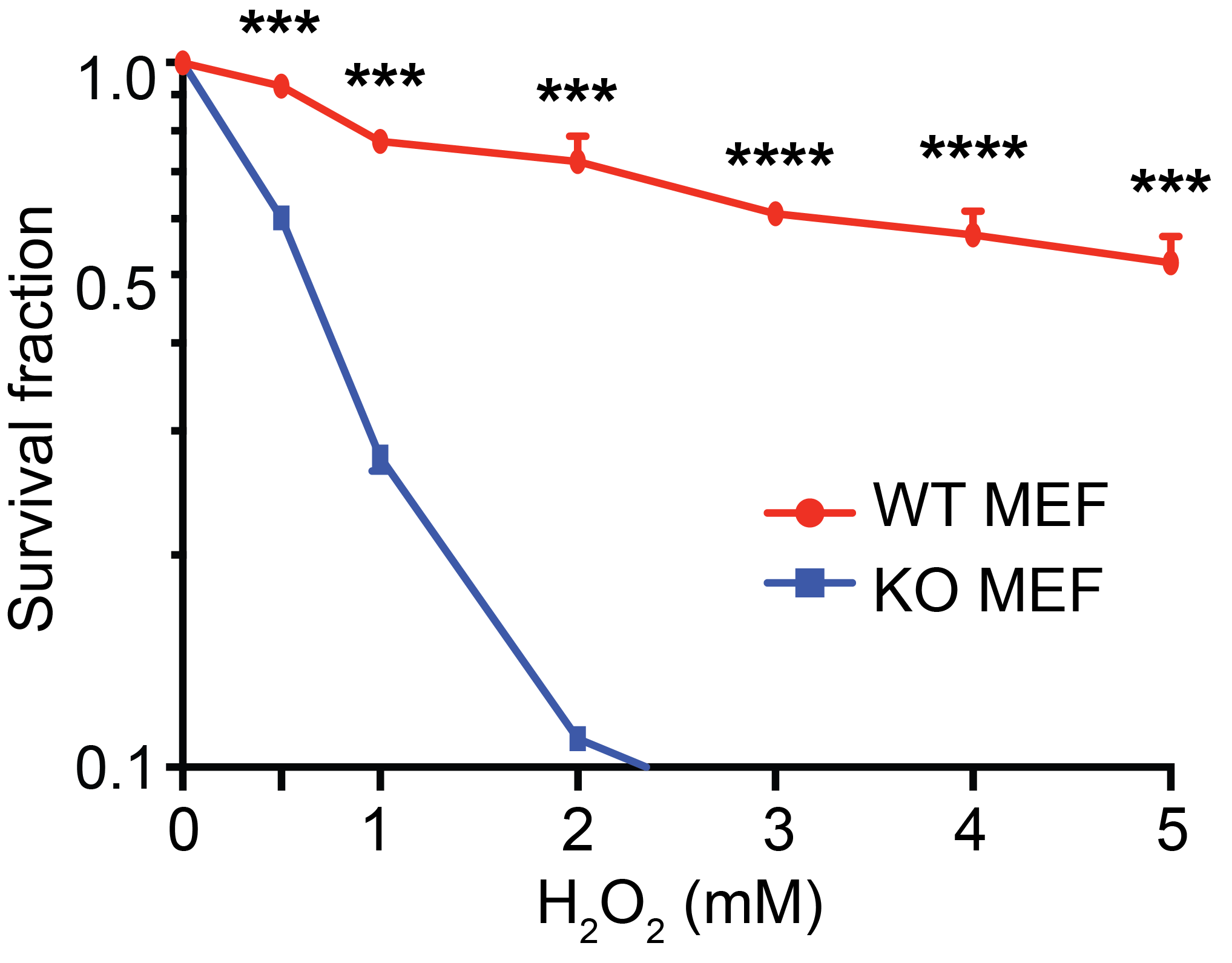

Supplement: S1 Fig — Survival fraction of wild-type (WT) and Sam68 KO MEFs 96 h post treatment with indicated concentrations of H2O2 for 15 min. Results are expressed as mean and SEM. ***, p < 0.001; ****, p < 0.0001 by Student’s t tests. Underlying data are shown in S1 Data. (TIF) [file pbio.1002543.s002.tif]

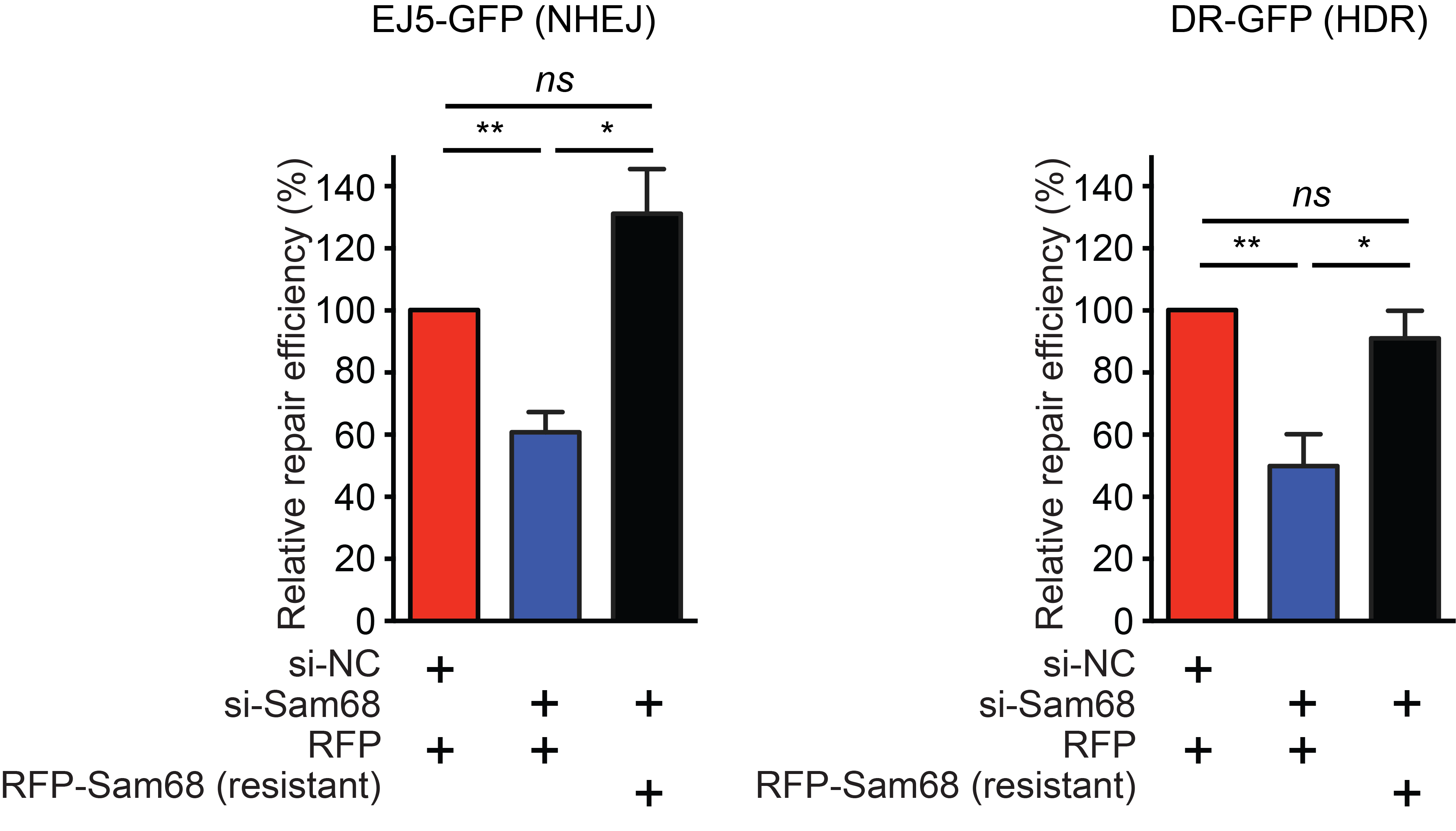

Supplement: S2 Fig — U2OS reporter cell lines, specifically designed to repair DNA damage through NHEJ and HDR, were transfected with nonspecific control (si-NC) or Sam68-specific (si-Sam68) siRNA. Forty-eight hours later, cells were transfected with siRNAs and RFP or siRNA-resistant RFP-Sam68, as indicated, together with (+) or without (−) I-SceI plasmid. Another 72 h later, cells were harvested for flow cytometric analyses of the DNA damage repair efficiency in the indicated reporter cell lines. The relative repair efficiency (normalized to si-NC, RFP, and I-SceI cotransfected cells) was quantified from three independent experiments. Results are expressed as mean and SEM. ns, nonsignificant difference; *, p < 0.05; **, p < 0.01; by Student’s t tests. Underlying data are shown in S1 Data. (TIF) [file pbio.1002543.s003.tif]

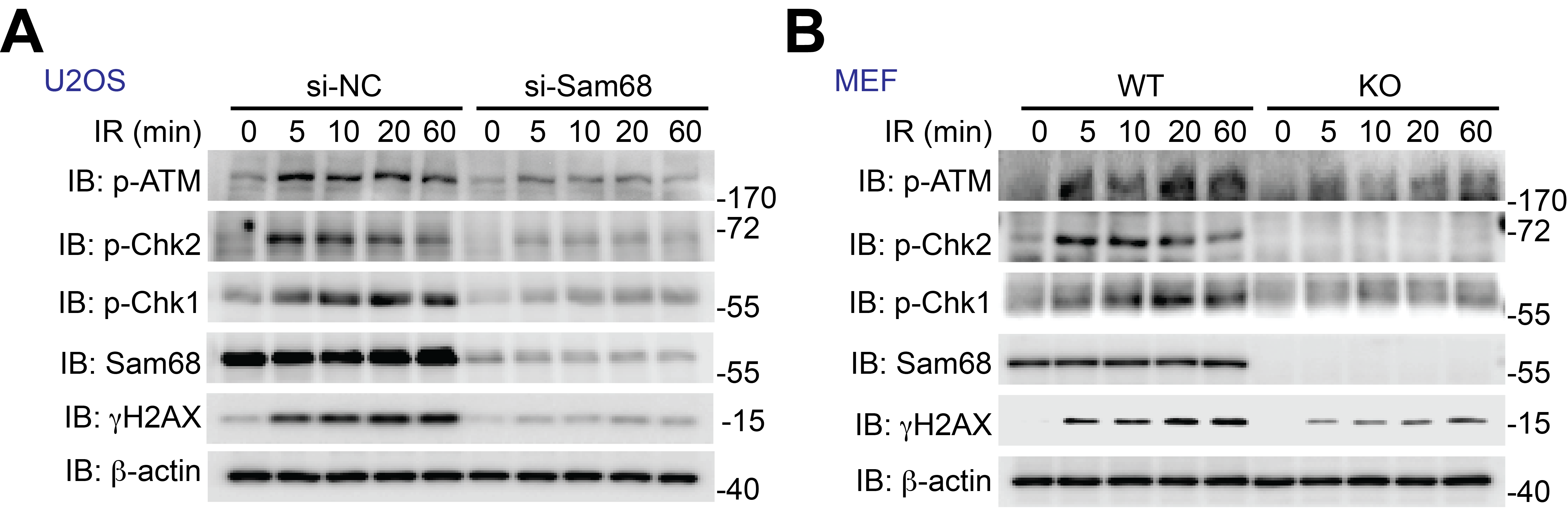

Supplement: S3 Fig — (A, B) U2OS cells transiently transfected with si-NC or si-Sam68 siRNA (A) or WT and Sam68 KO MEFs (B) were treated with 4 Gy of γ-irradiation (IR). Whole cell lysates were derived at indicated time points following IR and immunoblotted for indicated proteins, with β-actin as a loading control. (TIF) [file pbio.1002543.s004.tif]

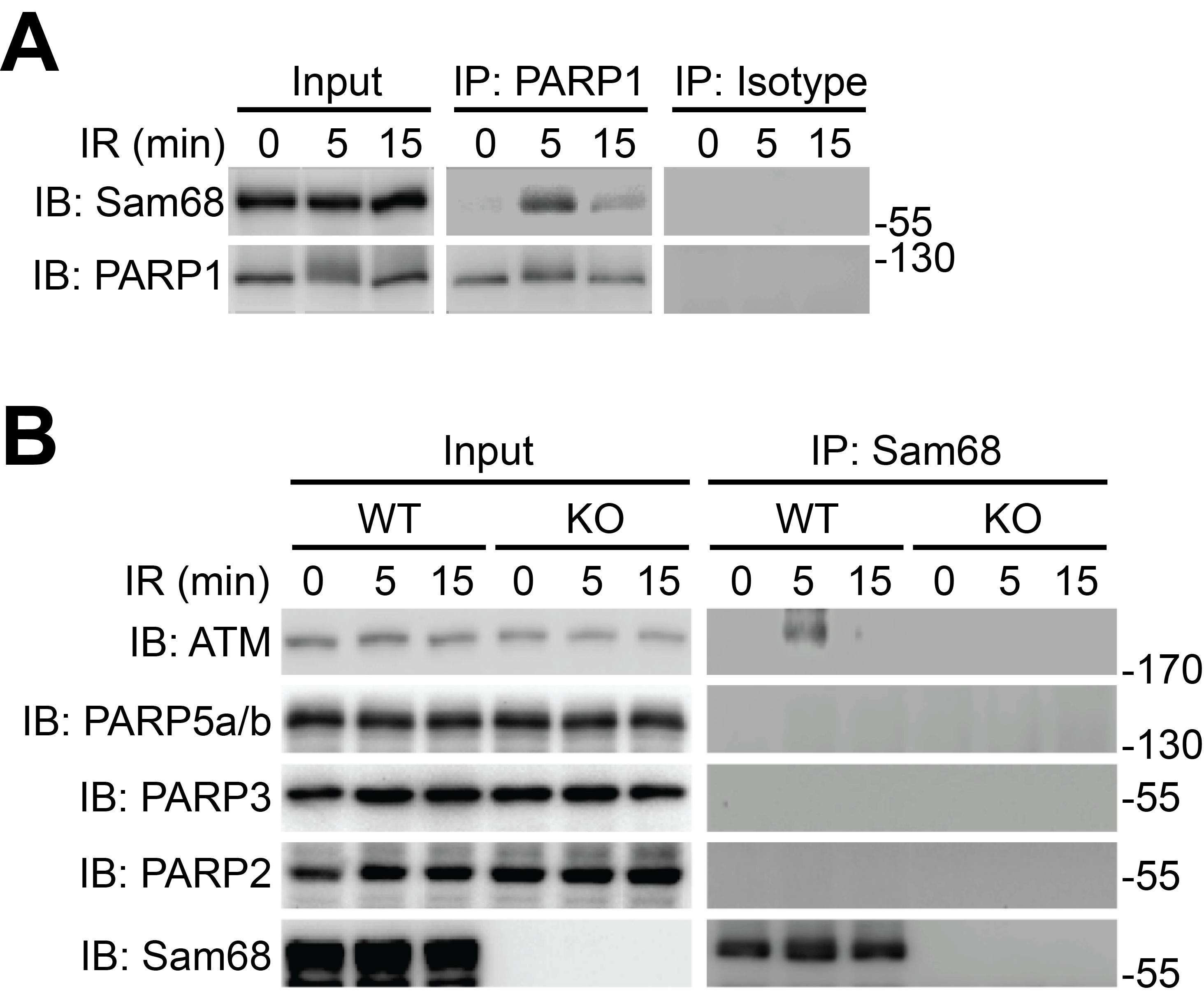

Supplement: S4 Fig — (A) Coimmunoprecipitation showing the inducible Sam68-PARP1 interaction. WT MEFs were γ-irradiated at 10 Gy, and whole cell lysates (Input) derived at indicated time points post irradiation were immunoblotted directly or after immunoprecipitated (immunoprecipitation, IP) with PARP1 or isotype control antibody for indicated proteins. (B) WT and Sam68 KO MEFs were γ-irradiated as in (A). Whole cell lysates (Input) derived at the indicated periods post IR were immunoblotted directly or after immunoprecipitated with Sam68 antibody for the indicated proteins. (TIF) [file pbio.1002543.s005.tif]

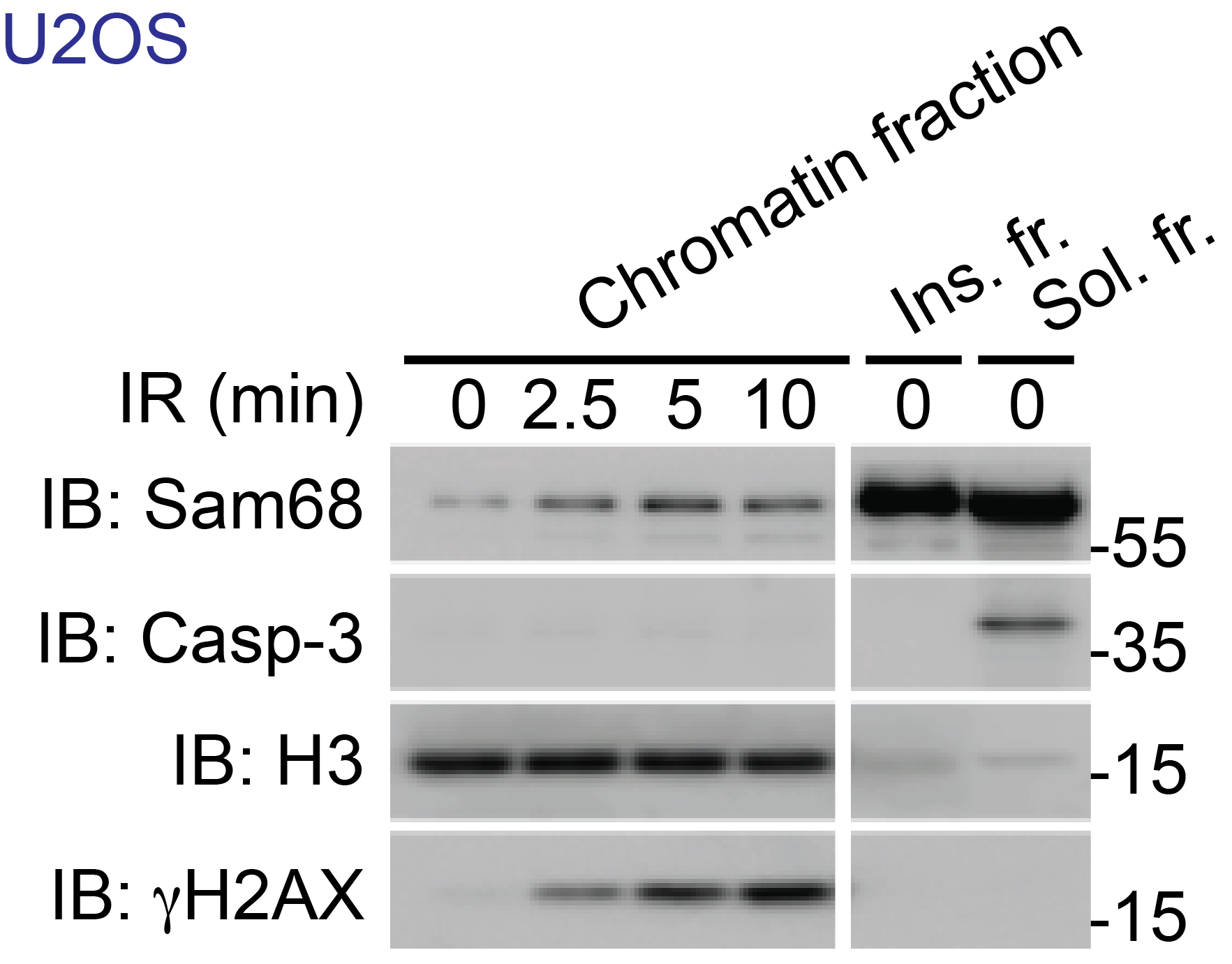

Supplement: S5 Fig — U2OS cells were γ-irradiated at 10 Gy, and the chromatin, soluble (Sol. fr.), and insoluble (Ins. fr.) subcellular fractions were derived at indicated time points following IR and immunoblotted for indicated proteins. Casp-3, Caspase-3; H3, Histone H3. (TIF) [file pbio.1002543.s006.tif]

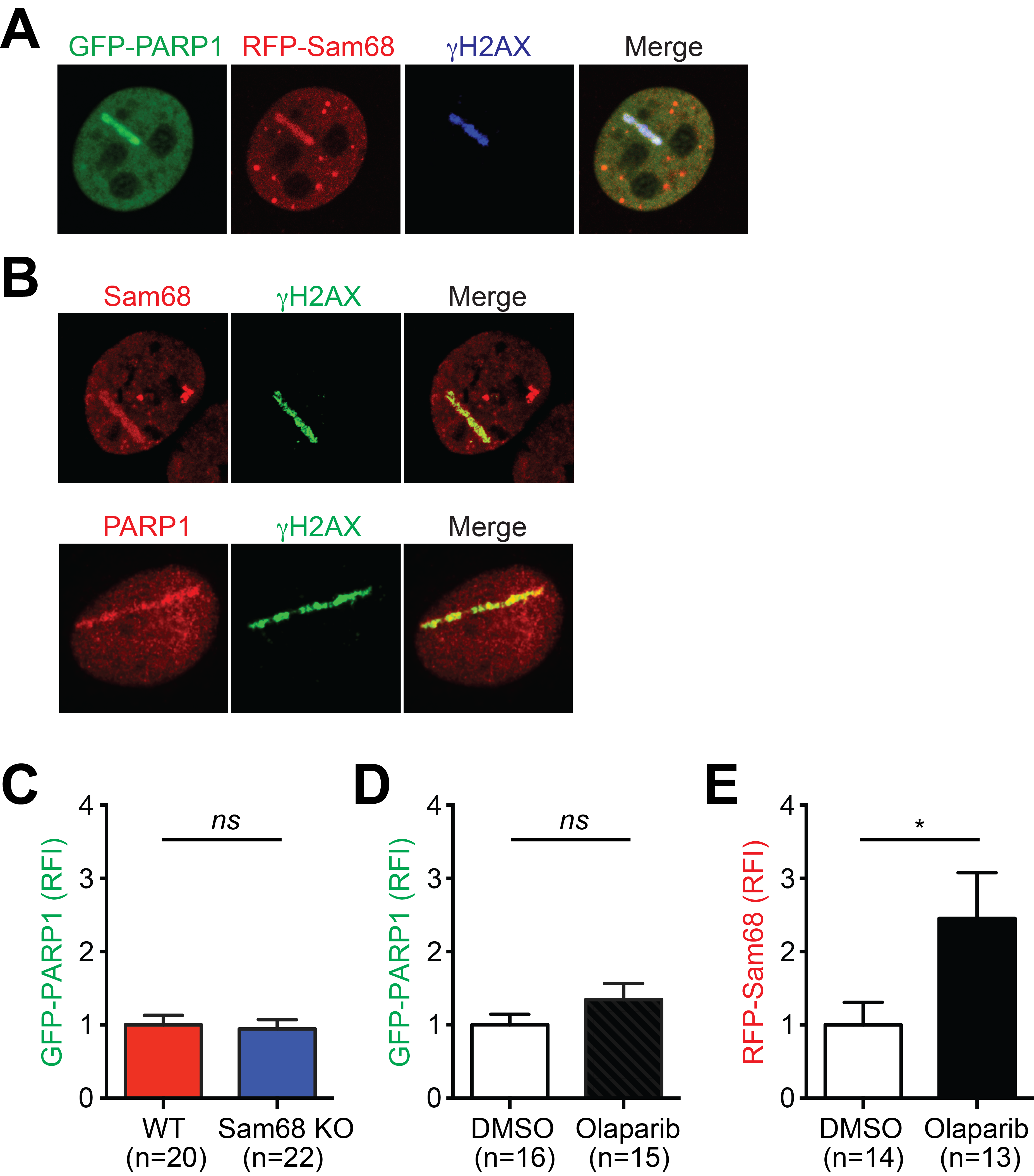

Supplement: S6 Fig — (A) Sam68 KO MEFs transiently expressing RFP-Sam68 together with GFP-PARP1 were subjected to laser microirradiation (Micro-IR). Cells were fixed at 1 min post Micro-IR and stained for endogenous γH2AX. Shown are fluorescence micrographs of RFP-Sam68, GFP-PARP1, and endogenous γH2AX at DNA damage foci. (B) Immunofluorescence micrographs of endogenous Sam68, PARP1, and γH2AX in WT MEFs at 1 min post Micro-IR. (C) WT and Sam68 KO MEFs expressing GFP-PARP1 were subjected to Micro-IR and the increase in relative fluorescence intensity (RFI) of GFP-PARP1 at damage foci ~10 s post Micro-IR versus pre-Micro-IR in WT, and Sam68 KO MEFs were graphed, normalized to WT controls. (D) WT MEFs expressing GFP-PARP1 were pretreated with DMSO or 10 μM of Olaparib for 90 min, followed by Micro-IR. The increase in RFI of GFP-PARP1 at damage foci ~10 s post Micro-IR versus pre-Micro-IR in DMSO- or Olaparib-treated cells was graphed, normalized to DMSO controls. (E) Sam68 KO MEFs expressing RFP-Sam68 were pretreated with DMSO or 10 μM of Olaparib for 90 min, followed by Micro-IR. The increase in RFI of RFP-Sam68 at damage foci ~10 s post Micro-IR versus pre-Micro-IR in DMSO- or Olaparib-treated cells was graphed, normalized to DMSO controls. Results in (C–E) are expressed as mean and SEM. ns, nonsignificant difference; *, p < 0.05 by Student’s t tests. Underlying data are shown in S1 Data. (TIF) [file pbio.1002543.s007.tif]

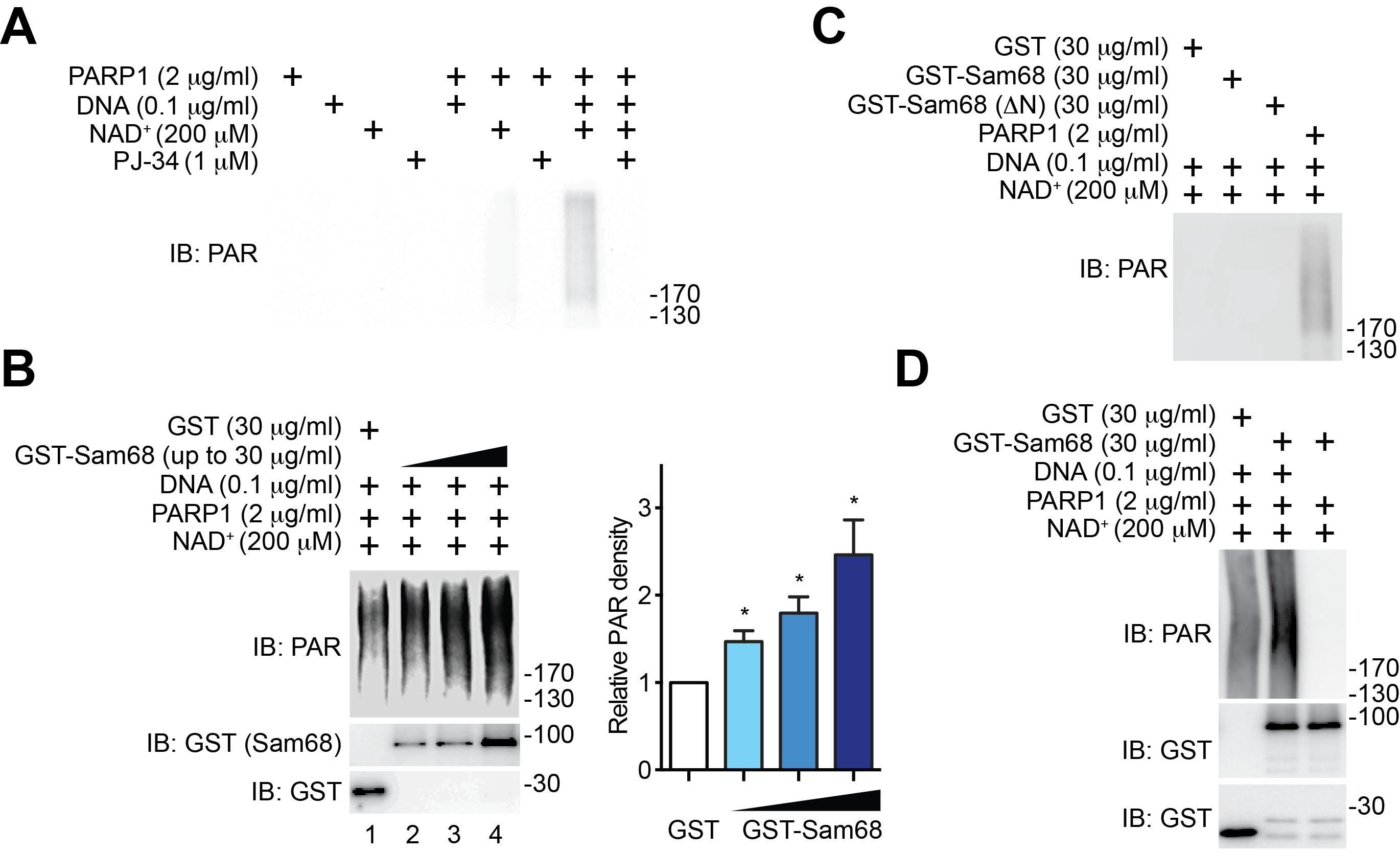

Supplement: S7 Fig — (A) Recombinant PARP1 protein was incubated in reaction buffer in the presence and absence of damaged DNA, NAD+, and PARP inhibitor PJ-34, as indicated. The reaction mixture was separated by SDS-PAGE and subjected to immunoblotting with the PAR antibody. (B) GST control or increasing amount of GST-Sam68 recombinant proteins were incubated with recombinant PARP1 protein in reaction buffer containing damaged DNA and NAD+, with indicated final concentrations. The reaction mixture was separated by SDS-PAGE and subjected to IB with the PAR and GST antibodies. Right, quantification of relative PARP1 activity based on PAR band density (normalized to the GST control), summarized from three independent experiments. Results are expressed as mean and SEM. *, p < 0.05 by Student’s t tests. (C) The indicated recombinant proteins were incubated in reaction buffer containing damaged DNA and NAD+. The reaction mixture was separated by SDS-PAGE and subjected to IB with the PAR antibody. (D) The indicated recombinant proteins were incubated in reaction buffer containing NAD+ in the presence and absence of damaged DNA. The reaction mixture was separated by SDS-PAGE and subjected to IB with the PAR and GST antibodies. Underlying data are shown in S1 Data. (TIF) [file pbio.1002543.s008.tif]

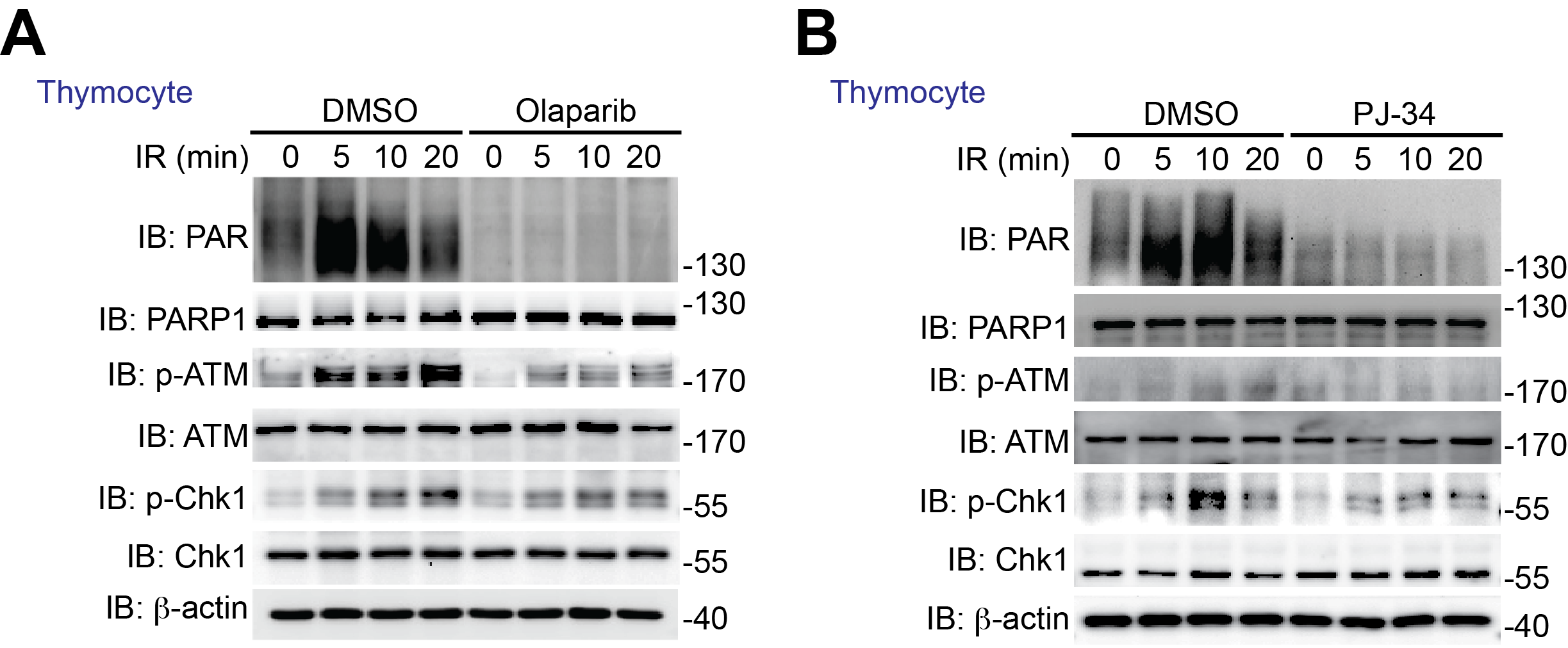

Supplement: S8 Fig — (A, B) Sam68+/- thymocytes pretreated with Olaparib (20 μM) (A), PJ-34 (20 μM) (B), or DMSO for 2 h were γ-irradiated at 4 Gy, and whole cell lysates were derived at indicated time points and immunoblotted for the indicated proteins, with β-actin as a loading control. (TIF) [file pbio.1002543.s009.tif]

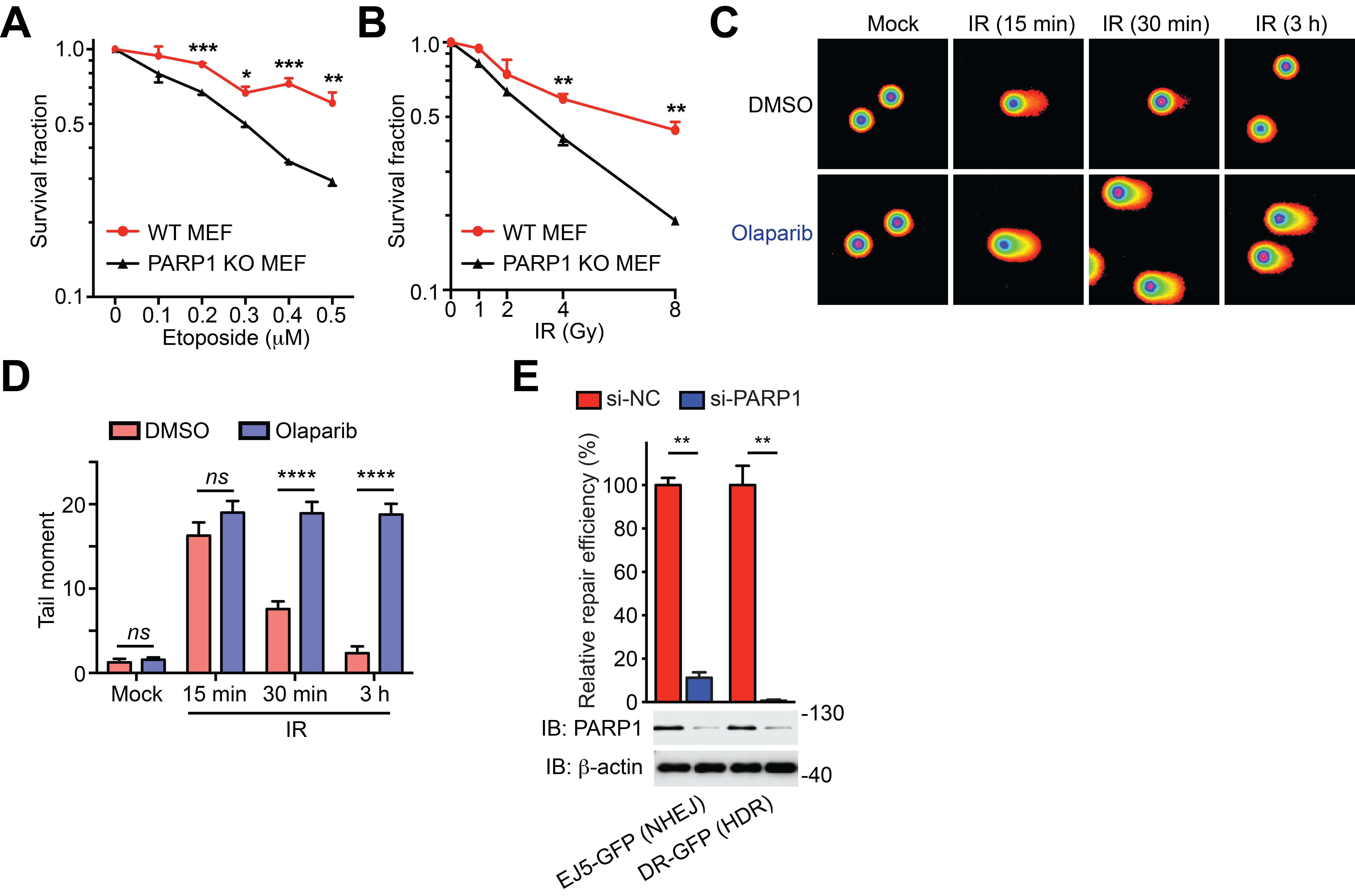

Supplement: S9 Fig — (A, B) Survival fraction of WT and PARP1 KO MEFs 96 h post treatment with indicated concentrations of etoposide for 20 h (A) or indicated doses of IR (B). (C) WT thymocytes were pretreated with DMSO or 20 uM of Olaparib, followed by 4 Gy of IR or mock-irradiation. Cells were harvested at the indicated time points post IR and subjected to alkali comet assay. Shown are representative microphotographs. (D) Quantification of tail moments in (C), with summarized data from 40–60 cells within 15 random fields for each time point. (E) U2OS reporter cell lines, specifically designed to repair DNA damage through NHEJ and HDR, were transfected with si-NC or PARP1-specific (si-PARP1) siRNA, together with (+) or without (−) I-SceI plasmid, or GFP control. Seventy-two hours later, cells were harvested for flow cytometric analyses of the effect of PARP1 knockdown on DNA damage repair efficiency in the indicated reporter cell lines. The relative repair efficiency (normalized to si-NC and I-SceI cotransfected cells) was quantified from three independent experiments. The PARP1 knockdown efficiency was examined by immunoblotting, with β-actin as a loading control, in the indicated reporter cell lines (bottom). Results in A–B and D–E are expressed as mean and SEM. ns, nonsignificant difference; *, p < 0.05; **, p < 0.01; ***, p < 0.001; ****, p < 0.0001 by Student’s t tests. Data are representative of two independent experiments. Underlying data are shown in S1 Data. (TIF) [file pbio.1002543.s010.tif]

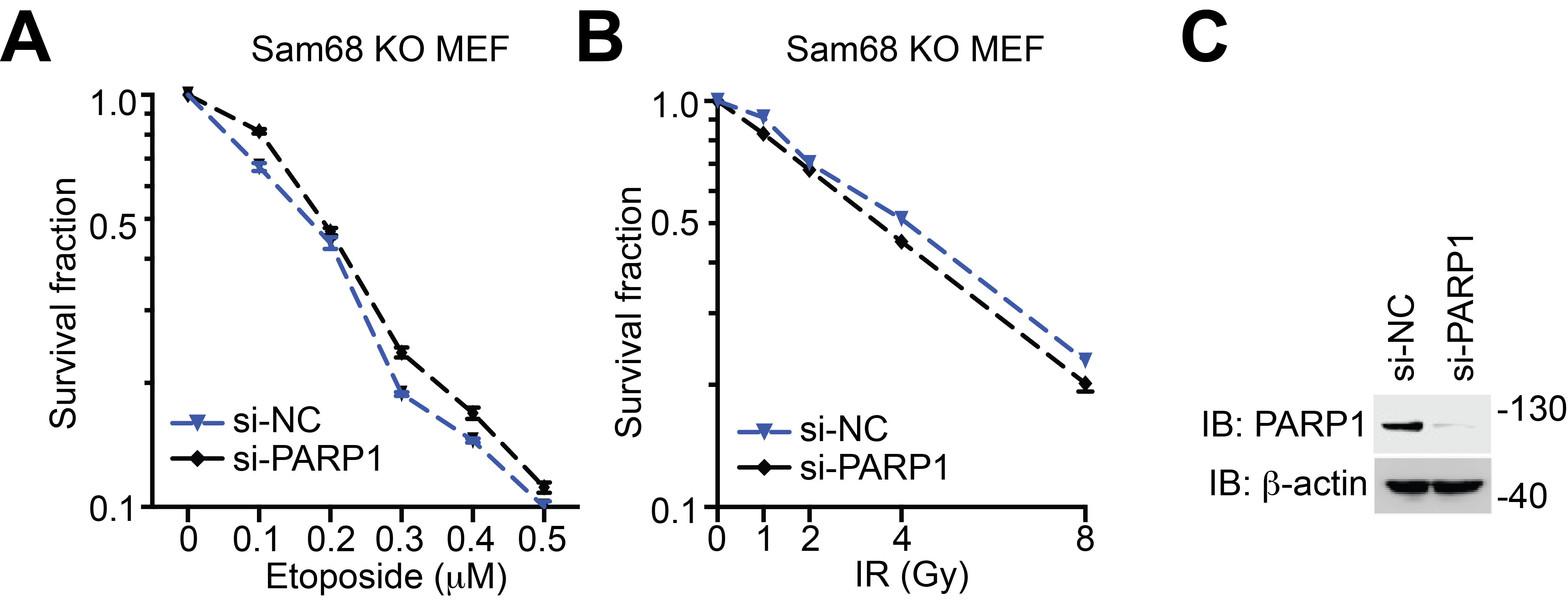

Supplement: S10 Fig — (A, B) Survival fraction of Sam68 KO MEFs silenced with si-NC or si-PARP1 siRNA 96 h post treatment with indicated concentrations of etoposide for 20 h (A) or indicated doses of IR (B). (C) The PARP1 knockdown efficiency was examined by immunoblot, with β-actin as a loading control, in Sam68 KO MEFs at 96 h after siRNA transfection. Underlying data are shown in S1 Data. (TIF) [file pbio.1002543.s011.tif]

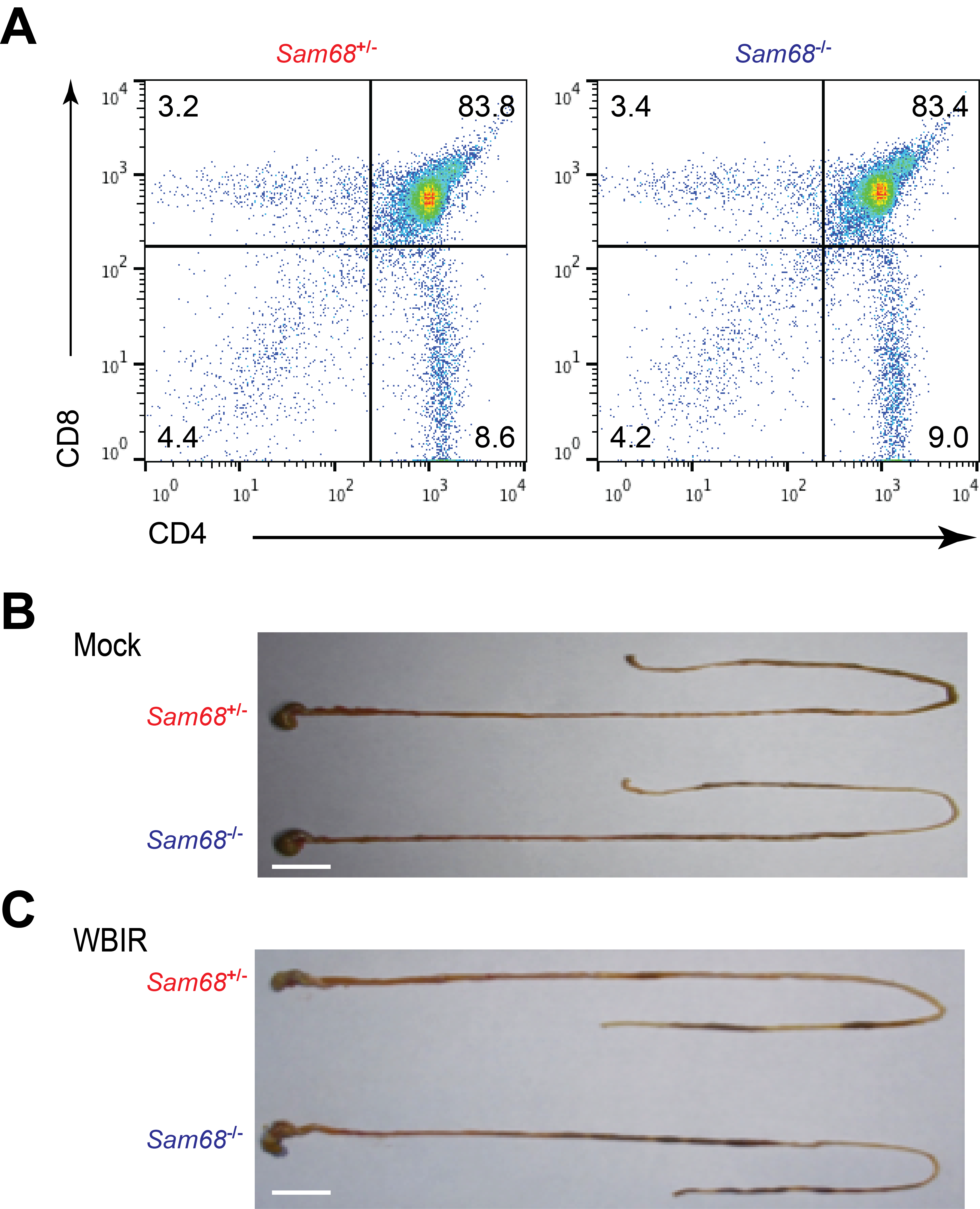

Supplement: S11 Fig — (A) Representative flow cytometry analysis of indicated immune cell subpopulations in thymocytes derived from naïve Sam68+/- and Sam68-/- mice. (B, C) Photographs of small intestines collected from Sam68+/- and Sam68-/- mice at 14 d post mock-irradiation (B) or WBIR (C). Scale bars, 2 cm. (TIF) [file pbio.1002543.s012.tif]
